# OH-Initiated Photooxidation of Gas-Phase Atmospherically Relevant Monoterpene-Derived Organic Nitrates

**DOI:** 10.1021/acs.est.5c07271

**Published:** 2025-12-24

**Authors:** Yuchen Wang, Yu Kang Xie, Masayuki Takeuchi, Gamze Eris, Nga L. Ng

**Affiliations:** † College of Environmental Science and Engineering, 12569 Hunan University, Changsha, Hunan 410082, China; ‡ School of Chemical and Bimolecular Engineering, 1372 Georgia Institute of Technology, Atlanta, Georgia 30332, United States; § School of Civil and Environmental Engineering, 1372 Georgia Institute of Technology, Atlanta, Georgia 30332, United States; ∥ School of Earth and Atmospheric Sciences, 1372 Georgia Institute of Technology, Atlanta, Georgia 30332, United States

**Keywords:** monoterpene, organic nitrate, organonitrate, monoterpene nitrate, nitrogen budget, NO_*x*_ recycling, secondary organic aerosol, ozone

## Abstract

Monoterpene-derived organic nitrates (MT-ONs) can influence NO_*x*_ recycling, secondary organic aerosol, and ozone formation. While OH-initiated photooxidation is considered a sink for MT-ONs, the rate constants and mechanisms remain poorly constrained. We investigate the gas phase photooxidation of three synthetic ONs derived from α-pinene, β-pinene, and d-limonene (3°_ApHN, 1°_BpHN, and 2°_LmHN) through chamber experiments. The photooxidation rate constants for MT-ONs range from (5.7 ± 0.5) to (11.0 ± 1.5) × 10^–11^ cm^3^ molecule^–1^ s^–1^, with corresponding lifetimes of 1.7–3.2 h under ambient OH concentrations. If we consider that products without a nitrooxy group (CHO) represent MT-ONs undergoing photooxidation to form NO_*x*_ or nitric acid, our measurements suggest that up to 85%, 49%, and 23% of 3°_ApHN, 2°_LmHN, and 1°_BpHN, respectively, contribute to NO_*x*_ recycling. The prevalence of CHO products is influenced by the molecular structure of MT-ONs and different peroxy radical chemistry. These results are different from prior studies that suggest that photooxidation leads to the complete degradation of MT-ONs into NO_*x*_ or nitric acid. We propose photooxidation mechanisms, highlighting both OH addition and H abstraction as important processes. This study provides fundamental data to evaluate the contributions of MT-ON photooxidation to NO_*x*_ recycling and offers new insights into the broader implications of ON chemistry in the atmosphere.

## Graphical abstract

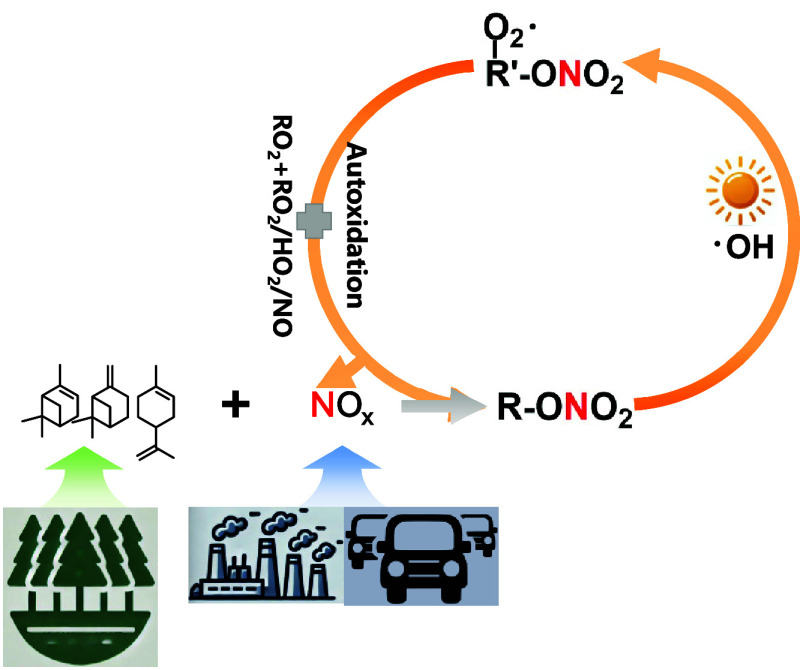

## 1 Introduction

Monoterpenes are a major class of biogenic volatile organic compounds (BVOCs) in the atmosphere, with large emissions on regional and global scales.^1,2^ They have been shown to contribute substantially to global secondary organic aerosol (SOA) and ozone budget owing to their high reactivity with atmospheric oxidants and subsequent formation of ozone.^3–6^ The conversion of monoterpenes to SOA and their impact on the ozone budget can be largely enhanced in areas with high anthropogenic emissions.^6–11^ One such pathway involves the formation of monoterpene-derived organic nitrates (MT-ONs) in the presence of NO_*x*_.^9–26^ MT-ONs have been identified as major oxidized products of monoterpenes, as evidenced by both chamber experiments^10–23^ and ambient field measurements.^9,26–28^ Laboratory studies have demonstrated that MT-ONs can be generated through photochemical reactions initiated by hydroxyl radical (OH) with NO, with molar yields of up to 40%.^24,25^ Additionally, dark chemical reactions initiated by nitrate radical (NO_3_) lead to substantial formation of MT-ONs, with molar yields ranging from 28% to 74%.^10–21^ Ambient field measurements have shown that MT-ONs are prevalent in areas with significant biogenic–anthropogenic interactions, where oxidation of monoterpenes contributes to a large fraction of SOA observed in the southeastern U.S.^9,26–29^ Therefore, it is important to understand the role of MT-ONs in NO_*x*_ recycling, ozone formation, and SOA production.

Once formed, the atmospheric impact of MT-ONs largely depends on whether they serve as a permanent sink or a temporary reservoir for NO_*x*_.^30^ This is contingent upon the kinetic fate of the MT-ONs to either retain or release NO_*x*_ through subsequent reactions. Results from field and modeling studies suggest that the ambient lifetime of ON spans just a few hours.^26,31–33^ Photolysis and hydrolysis have been shown to be the major subsequent reactions for MT-ONs, serving as sinks for gas- and particle-phase MT-ONs, respectively.^34–37^ Photolysis recycles NO_*x*_ through direct photolytic cleavage of the nitrooxy group in MT-ONs. The ambient photolysis lifetime of MT-ONs can be as short as 0.43 h for those generated from nitrate radical oxidation of monoterpenes^99^ or 2 h for synthetic monoterpene hydroxy nitrates, followed by formation of ozone.^38^ Condensed phase hydrolysis has been identified as an important sink for both MT-ONs and NO_*x*_, as nitric acid generated from the hydrolysis of MT-ONs in aqueous aerosol can be removed through dry and wet deposition.^36,39^ The significance of hydrolysis in processing MT-ONs largely depends on their hydrolysis lifetimes. Studies using bulk solutions have demonstrated that the hydrolysis lifetime of particle-phase MT-ONs can vary largely, ranging from several minutes to no observed hydrolysis over 7 days.^36,39^ This variability is controlled by the molecular structure of the MT-ONs.^36,39^

Although there is no prior study on OH radical initiated photooxidation of MT-ONs, photooxidation is considered the major sink for short-chain ONs with 3 to 5 carbon atoms, particularly those with specific molecular structures such as double bonds. Previous studies have shown that the addition of OH radical to the double bond in ONs occurs much faster than H abstraction, with lifetimes ranging from 1.7 to 5.0 h for addition and from 64 to 712 h for abstraction ([OH] = 1.5 × 10^6^ molecules cm^–3^).^40–44^ Considering that the OH radical is the most effective oxidant for daytime chemistry,^45^ maintaining relatively high daytime concentrations on the order of 10^6^ molecules cm^–3^, photooxidation has the potential to be a major sink for MT-ONs. However, major knowledge gaps exist with respect to photooxidation rate constants, influence of molecular structure, and mechanisms for MT-ONs photooxidation, limiting our understanding of monoterpene chemistry.

In this work, we investigate the photooxidation of MT-ONs, using three synthetic ONs with hydroxyl group derived from α-pinene, β-pinene, and d-limonene as model compounds. These ONs are formed in the atmosphere through OH-oxidation in the presence of NO_*x*_ and/or NO_3_ oxidation of α-pinene, β-pinene, and limonene.^23,46,47^ We systematically conduct a series of chamber experiments under different peroxy radical (RO_2_) fates (RO_2_ + NO dominant and RO_2_ + RO_2_/HO_2_ dominant),^48^ to determine the photooxidation rate constants and to characterize the chemical composition of major compounds in both gas and particle phases resulting from the photooxidation of MT-ONs. Based on the major compounds identified using a high-resolution time-of-flight chemical-ionization mass spectrometer coupled with the Filter Inlet for Gases and Aerosols (FIGAERO–CIMS), we propose mechanisms for the photooxidation of MT-ONs. This comprehensive chamber study provides fundamental data to better understand the photochemical fates of MT-ONs and their roles as NO_*x*_ reservoirs/sinks, as well as their potential influence on NO_*x*_ recycling and SOA formation.

## 2 Experimental Section

### 2.1 Sample Preparation

A total of three MT-ONs is synthesized in this study. The synthesis of MT-ONs follows the strategies reported in our previous study.^35^ Briefly, β-pinene (Bp) and d-limonene (Lm) directly react with *N*-bromosuccinimide^49,50^ and undergo halohydrination to generate bromohydrins. Subsequent nucleophilic substitution of the bromohydrins derived from β-pinene and limonene with silver nitrate (AgNO_3_)^51–54^ yields primary β-pinene hydroxynitrate (1°_BpHN) and secondary limonene hydroxynitrate (2°_LmHN), respectively. The epoxide-opening reaction^55–57^ of α-pinene (Ap) oxide with fuming nitric acid can directly produce tertiary α-pinene hydroxynitrate (3°_ApHN). The molecular structures of these MT-ONs are shown in Table 1. The purities of all synthetic MT-ONs are higher than 99% based on nuclear magnetic resonance (NMR) spectra except 3°_ApHN (∼98%). Pure standards are stored in a freezer (−20 °C) until use.

**Table 1:**
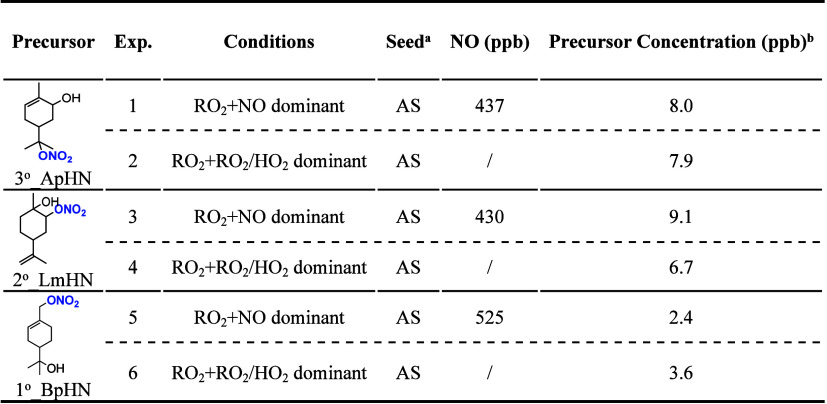
Summary of Experimental Conditions

a (NH_4_)_2_SO_4_ seed.

b Measured by TD-CAPS, with uncertainty = 0.6 ppb.

### 2.2 Environmental Chamber Experiments

Experiments are conducted in the Georgia Tech Environmental Chamber (GTEC) facility using the 12 m^3^ flexible Teflon chambers at 295 ± 3 K and ambient pressure.^19^ The chamber is flushed with zero air (Aadco, 747-14) prior to each experiment for at least 36 h. This ensures that the background concentrations of ozone, NO, and NO_2_ are less than 1 ppb. Experiments are performed under dry conditions (<3% RH).

Experiments are designed to explore the photooxidation of three synthetic MT-ONs (3°_ApHN, 2°_LmHN, and 1°_BpHN) under different peroxy radical chemistry regimes (RO_2_ + NO dominant or RO_2_ + RO_2_/HO_2_ dominant) with and without NO addition to the chamber. The various peroxy radical chemistry regimes are estimated using the Framework for 0-D Atmospheric Modeling (F0AM) under different NO concentrations.^19,21^ It is noted that the reaction rate constants for RO_2_ + RO_2_ in the Master Chemical Mechanism (MCM, version 3.3.1) are highly uncertain,^58–60^ therefore, RO_2_ + RO_2_ could also be important in experiments where RO_2_ + HO_2_ is expected to be dominant. Hence, we label the RO_2_ + HO_2_ dominant condition as RO_2_ + RO_2_/HO_2_ dominant. The experimental conditions are summarized in Table 1.

In RO_2_ + RO_2_/HO_2_ dominant experiments, 10 μL of MT-ON is transferred into a glass bulb, and zero air is passed over the MT-ON at a rate of 5 L min^–1^ to facilitate evaporation and introduction of the precursor into the chamber. The bulb is gently heated to accelerate the evaporation process. The initial concentrations of 3°_ApHN, 2°_LmHN, and 1°_BpHN are 7.9, 6.7, and 3.6 ppb, respectively. Seed particles are generated by atomizing a dilute ammonium sulfate solution (0.015 M) and are passed through a dryer before entering the chamber. The number and volume concentrations of seed aerosol are approximately 2 × 10^4^ cm^–3^ and 2 × 10^10^ nm^3^ cm^–3^, respectively. For each MT-ON, nucleation experiments are performed without seed particles to determine the density of organic aerosol. Cyclohexane (100 ppb) is also added to the chamber as the reference compound to track OH concentration. After that, H_2_O_2_ (2 ppm) is introduced into the reactor in the same manner as MT-ON and used as the photolytic source of OH radical. The experiments are initiated (time zero) by turning on the chamber black lights (Sylvania, 24922) approximately 30 min after the end of the H_2_O_2_ injection to ensure that the chamber content is well-mixed.

The procedure for RO_2_ + NO dominant experiments is the same except the additional injection of a desired amount of NO (430–525 ppb) to the chamber from a cylinder containing 500 ppm of NO (Matheson) after the H_2_O_2_ injection. Any ozone formed in the experiment is titrated by the added NO, hence there is no consumption of MT-ONs by ozone even though they have double bonds. The high level of NO can also suppress the RO_2_ + RO_2_ and RO_2_ + HO_2_ reactions.

### 2.3 Instrumentation

The concentrations of ozone and NO_*x*_ are continuously monitored using an ultraviolet absorption ozone monitor (Teledyne T400) and a chemiluminescence NO_*x*_ monitor (Thermo Fisher Scientific 42C), respectively. To monitor the decay of cyclohexane, we employ a gas chromatograph equipped with a flame ionization detector (GC–FID, Agilent). A thermal dissociation cavity attenuated phase shift spectroscopy (TD-CAPS) is used to measure the MT-ON concentration in the chamber.^34,61^ In brief, the instrument features three channels and a CAPS monitor specifically for measuring NO_2_. The reference channel records the background NO_2_ within the chamber at room temperature. The other two channels are equipped with quartz tube reactors heated to 653 and 473 K, respectively, to facilitate the quantitative conversion of nitrooxy groups from alkyl nitrates and peroxy nitrates into NO_2_. Each channel operates with a sampling duration of 2 min. In this study, the concentration of injected MT-ON in the chamber is determined by comparing the NO_2_ levels measured at 653 K and room temperature.

Particle size distributions and volume concentrations for particles under 1 μm (electrical mobility diameter) are recorded using a scanning mobility particle sizer (SMPS) set to low-flow mode (sheath flow of 2 L min^–1^), comprising a differential mobility analyzer (DMA, TSI 3080) and a condensation particle counter (CPC, TSI 3775). For bulk particle chemical composition analysis (including organics (Org), nitrate (NO_3_), sulfate (SO_4_), ammonium (NH_4_), and chloride (Chl)), we utilize a high-resolution time-of-flight aerosol mass spectrometer (HR-ToF-AMS, Aerodyne Research, Inc.). The operational principles and techniques of the HR-ToF-AMS are extensively documented in prior studies.^62^

Analysis of speciated oxidized organic species in both gas and particle phases is carried out using a FIGAERO–CIMS (Aerodyne Research, Inc.), which uses iodide (I^–^) as the reagent ion. Comprehensive methodological details are available in various publications.^20,21,29,37^ In brief, reagent ions are produced from a cylinder filled with a CH_3_I and dry N_2_ mixture (Airgas) and a polonium-210 source (NRD). The device samples atmospheric gases at a flow rate of 1.7 L min^–1^, while particles are collected on a polytetrafluoroethylene filter at a rate between 3 and 5 L min^–1^, based on aerosol mass concentrations. These collected organic species are subsequently volatilized by a controlled stream of heated nitrogen and analyzed by the CIMS. Data processing is conducted using Tofware v2.5.11, and all detected compounds are identified as iodide adducts.

## 3 Results and Discussion

### 3.1 Photooxidation Rate Constants of MT-ONs

The photooxidation rate constants of MT-ONs are determined from experiments conducted in the presence of NO. In all experiments, the progression of the reaction is monitored by observing the decay of the normalized C_10_H_17_NO_4_I^–^ signal (an ion cluster of I^–^ with MT-ON) using CIMS. The time profile of a typical photooxidation experiment of MT-ON is illustrated in Figure S1, using 3°_ApHN as an example. The high concentration of NO suppresses the ozone level through its reaction with ozone, preventing the ozonolysis of MT-ON. Therefore, photooxidation, photolysis, and vapor wall loss are the mechanisms responsible for the decay of MT-ON during irradiation (*k*_UV_). The rate constants for photolysis (*j*_chamber_) and vapor wall loss (*k*_vwl_) have been determined and reported in our previous work.^34^ During irradiation, since H_2_O_2_ is present in large excess compared to the MT-ON, the concentration of the OH radical can be considered constant. Considering that both the photolysis and vapor wall loss of MT-ON are first-order reactions, the photooxidation of MT-ON follows a pseudo-first-order rate equation, therefore the decay of MT-ON during irradiation can be described using pseudo-first-order rate equation (Figure 1a) as well. Correspondingly, the photooxidation rate constant (*k*_OH_) of MT-ON can be determined by the following equations:

$$\begin{array}{cc} -\frac{d\left[\mathrm{MT‐ON}\right]}{dt} & =k_{\mathrm{UV}}\times \left[\mathrm{MT‐ON}\right]\\ & =\left(k_{\mathrm{OH}}^{\prime }+j_{\mathrm{chamber}}+k_{\mathrm{vwl}}\right)\times \left[\mathrm{MT‐ON}\right]\\ & =\left(k_{\mathrm{OH}}\times [OH]+j_{\mathrm{chamber}}+k_{\mathrm{vwl}}\right)\times \left[\mathrm{MT‐ON}\right] \end{array}$$ 1

where *k*_OH_^′^ is the pseudo-first-order rate constant for the photooxidation of MT-ON, *k*_OH_ is the second-order rate constant for the photooxidation of MT-ON, *j*_chamber_ is the photolysis rate constant, *k*_vwl_ is the vapor wall loss rate constant, and *k*_UV_ is the slope by plotting ln­{[MT-ON]_*t*_/[MT-ON]_0_} as a function of time in the irradiation period. [OH] is the concentration of OH radical.

**Figure 1:**
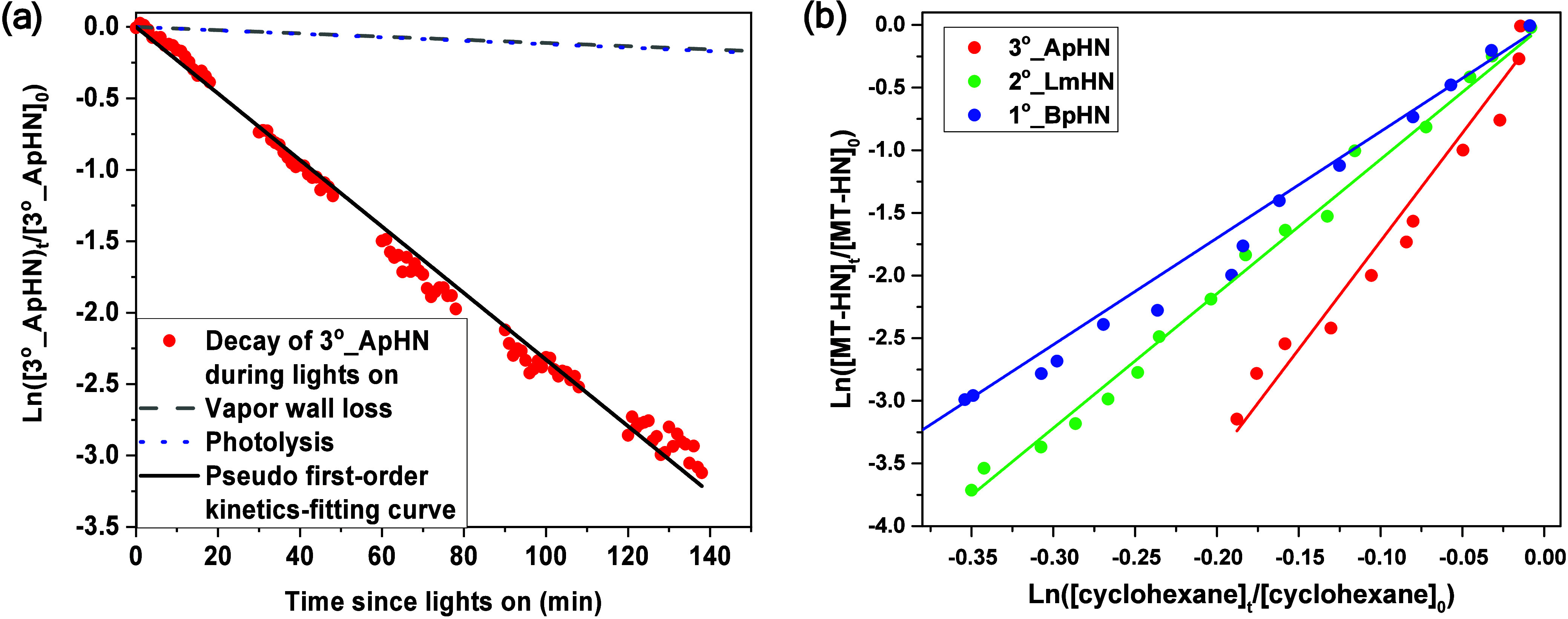
(a) Pseudo-first-order kinetics fitting curve for photooxidation of 3°_ApHN, alongside the first-order kinetics fitting curve for photolysis and vapor wall loss of 3°_ApHN. (b) Concentration ln–ln plot for the rate constant of each MT-ON relative to the rate constant of cyclohexane during irradiation.

Using the expression from the above equation, we can rewrite it as

$$k_{\mathrm{UV}}=\ln\frac{{\left[\mathrm{MT‐ON}\right]}_{t}}{{\left[\mathrm{MT‐ON}\right]}_{0}}=k_{\mathrm{OH}}^{\prime }+j_{\mathrm{chamber}}+k_{\mathrm{vwl}}=k_{\mathrm{OH}}\times \left[\mathrm{OH}\right]+j_{\mathrm{chamber}}+k_{\mathrm{vwl}}$$ 2

Therefore

$$k_{\mathrm{OH}}\times \left[\mathrm{OH}\right]=\ln\frac{{\left[\mathrm{MT‐HN}\right]}_{t}}{{\left[\mathrm{MT‐HN}\right]}_{0}}\times \frac{\left(k_{\mathrm{UV}}-j_{\mathrm{chamber}}-k_{\mathrm{vwl}}\right)}{k_{\mathrm{UV}}}$$ 3

where we define $\frac{\left(k_{\mathrm{UV}}-j_{\mathrm{chamber}}-k_{\mathrm{vwl}}\right)}{k_{\mathrm{UV}}}$ as the contribution of photooxidation to the overall decay of MT-ON during the irradiation period.

As a direct method for measuring the OH radical concentrations during chamber experiments is not available, the relative rate method is used to determine the photooxidation rate constants of MT-ONs with cyclohexane as the reference compound. The relative decays of MT-ONs and cyclohexane, presented in ln–ln scales, are depicted in Figure 1b. Consequently, the photooxidation rate constant of MT-ON can be calculated using the following equation (eq 4):

$$\frac{k_{\mathrm{OH}}}{k_{\mathrm{cyclohexane}}}=(\ln\frac{{\left[\mathrm{MT‐HN}\right]}_{t}}{{\left[\mathrm{MT‐HN}\right]}_{0}})\times \frac{\left(k_{\mathrm{UV}}-j_{\mathrm{chamber}}-k_{\mathrm{vwl}}\right)}{k_{\mathrm{UV}}}÷\left(\ln\frac{{\left[\mathrm{cyclohexane}\right]}_{t}}{{\left[\mathrm{cyclohexane}\right]}_{0}}\right)$$ 4

where *k*_cyclohexane_ is the photooxidation rate constant of cyclohexane (7.2 × 10^–12^ cm^3^ molecule^–1^ s^–1^ at 297 K; MCM, version 3.3.1). As shown in Figure 1b, the plot of ln $\frac{{\left[\mathrm{MT‐HN}\right]}_{t}}{{\left[\mathrm{MT‐HN}\right]}_{0}}$ versus ln $\frac{{\left[\mathrm{cyclohexane}\right]}_{t}}{{\left[\mathrm{cyclohexane}\right]}_{0}}$ is linear with an intercept of zero. The $\frac{k_{\mathrm{OH}}}{k_{\mathrm{cyclohexane}}}$ values are reported in Table S1. In addition, based on the decay rate of cyclohexane and its known OH reaction rate constant, the estimated OH concentrations ranged from 3.1 × 10^6^ to 4.6 × 10^6^ molecules cm^–3^.

The photooxidation rate constant for each MT-ON is calculated by multiplying the slope of the linear fit with the photooxidation rate constant of cyclohexane. The photooxidation rate constants are determined to be *k*_OH_ = (11.0 ± 1.5) × 10^–11^ cm^3^ molecule^–1^ s^–1^ for 3°_ApHN, (7.2 ± 0.4) × 10^–11^ cm^3^ molecule^–1^ s^–1^ for 2°_LmHN, and (5.7 ± 0.5) × 10^–11^ cm^3^ molecule^–1^ s^–1^ for 1°_BpHN. The uncertainties are calculated by propagation of statistical errors associated with rate constants for the vapor wall loss, photolysis, irradiation, and slope of concentration ln–ln plot. When considering an ambient relevant OH concentration as 1.5 × 10^6^ molecules cm^–3^, the corresponding photooxidation lifetimes are 1.7, 2.6, and 3.2 h for 3°_ApHN, 2°_LmHN, and 1°_BpHN, respectively. The photooxidation rate constants and lifetimes are summarized in Table 2. The MT-ONs photooxidation rate constants determined in this study are of the same order of magnitude as those for isoprene-derived ONs with the double bond, but are approximately 1–2 orders of magnitude faster than those for carbonyl nitrates without a double bond.^41,42^ The presence of a double bond is the controlling factor for enhancing the photooxidation rate constants of ONs, consistent with previous findings that OH addition to a double bond occurs much faster than H abstraction.^63^

**Table 2:**
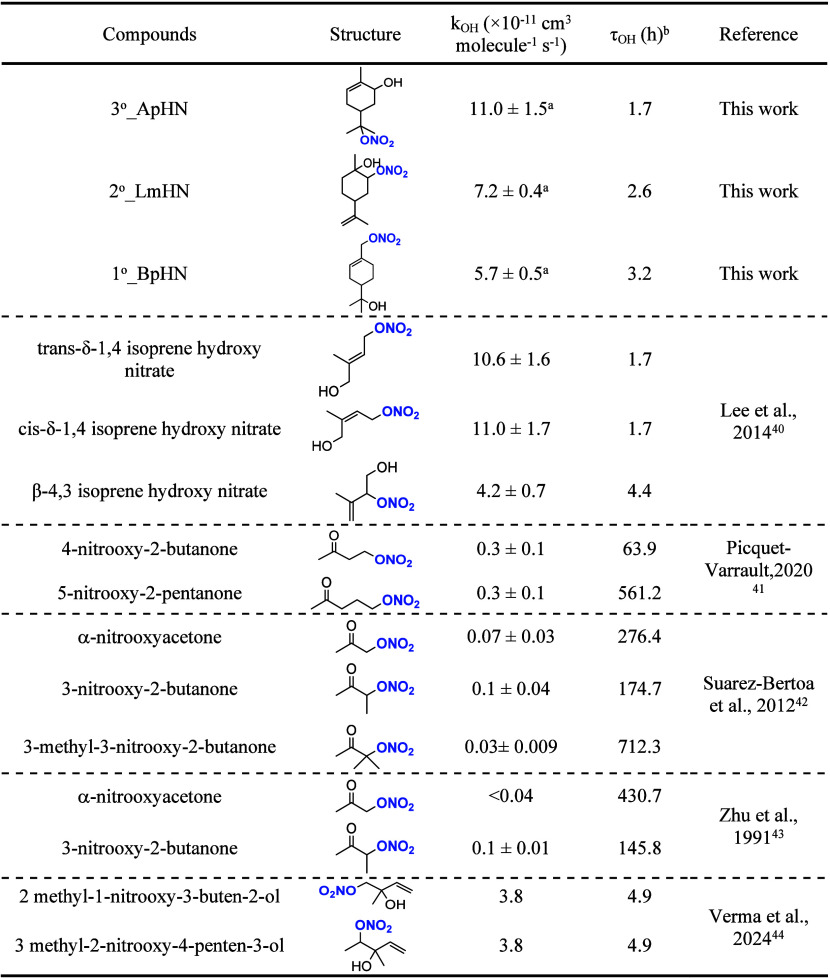
OH-Initiated Photooxidation Rate Constants of ONs in This Study and in the Literature

a The uncertainties are propagated from the statistical errors associated with the parameters in Table S1.

b [OH] = 1.5 × 10^6^ molecules cm^–3^.

We further compare the measured MT-ONs photooxidation rate constants from this study with theoretical predictions derived from structure–activity relationships (SARs). Based on parametrizations from Kwok and Atkinson,^63^ the theoretical *k*_OH_ values are 9.0 × 10^–11^ cm^3^ molecule^–1^ s^–1^ for 3°_ApHN, 5.5 × 10^–11^ cm^3^ molecule^–1^ s^–1^ for 2°_LmHN, and 4.4 × 10^–11^ cm^3^ molecule^–1^ s^–1^ for 1°_BpHN. These values are smaller than our measurements by a factor of 1.1–1.3 even when considering uncertainty. The smaller rate constants obtained from SARs suggest that the functional group contribution factors at the adjacent position of the double bond in substituted alkenes differs between this work and previous studies. Functional group contribution factors in SARs quantify how specific functional groups enhance or reduce the base reactivity of a molecular substructure when estimating reaction rate constants. In this work, we propose the functional group contribution factor for nitrooxy group, *F*(−CH_2_ONO_2_), to be 0.6 ± 0.2 by comparing the experimental and theoretical *k*_OH_ values of 1°_BpHN. This suggest that the nitrooxy group is less deactivating than previously reported by Kwok and Atkinson^63^ who estimated *F*(−CH_2_ONO_2_) as 0.5 based on a comprehensive summary of experimental rate constant data. On the other hand, Lee et al.^40^ propose a functional group contribution factor of 0.8, which falls within the uncertainty range of our estimate and is therefore consistent with our findings. Similarly, by comparing the measured and theoretical *k*_OH_ of 3°_ApHN, we propose a functional group contribution factor of hydroxyl group [*F*(−CHOH)] to be 1.2 ± 0.2, which is higher than in Kwok and Atkinson^63^ [*F*(−CHOH) = 1.0] and in Pfrang et al.,^64^ [*F*(−CHOH) = 0.7]. Our work provides new insights for the functional group contribution factors in SARs calculations, allowing for more accurate predictions of the rate constants for ambient MT-ONs, which are highly functionalized.

### 3.2 Chemical Analysis of Photooxidation Products and SOA Formation

The gas- and particle-phase products formed from the photooxidation of 3°_ApHN, 2°_LmHN, and 1°_BpHN are detected by the FIGAERO–CIMS. In this work, we introduce cyclohexane as a reference compound, which may result in the formation of compounds containing six or fewer carbon atoms. The mass spectra (normalized to the most abundant species) of all gas- and particle-phase products measured by the FIGAERO–CIMS at peak SOA mass concentrations in 3°_ApHN, 2°_LmHN, and 1°_BpHN experiments are shown in Figure 2 and Figures S2 and S3, respectively. As the products of monoterpene oxidation typically contain at least seven carbon atoms, we only regard products with seven or more carbon atoms (C > 7) as major products. In addition, for a species to be considered a major product, its normalized signal must also be greater than 0.1 relative to the compound with the highest detected signal. Using ozonolysis rate constants obtained from separate chamber experiments reported in our previous work^34^ (Table S3), the contribution of ozonolysis to the measured rates ranges from 3% to 8% under RO_2_ + RO_2_/HO_2_ conditions. Given the relatively small contribution of ozonolysis under these conditions, the products formed from ozonolysis are not discussed in this study. Figure 3 and Figures S4 and S5 present the corresponding normalized signal distributions of major gas- and particle-phase compounds categorized into three groups: CHO, CHON, and CHON_2_. CHO compounds are those without any nitrooxy groups. CHON and CHON_2_ compounds contain one and two nitrooxy groups, respectively.

**Figure 2:**
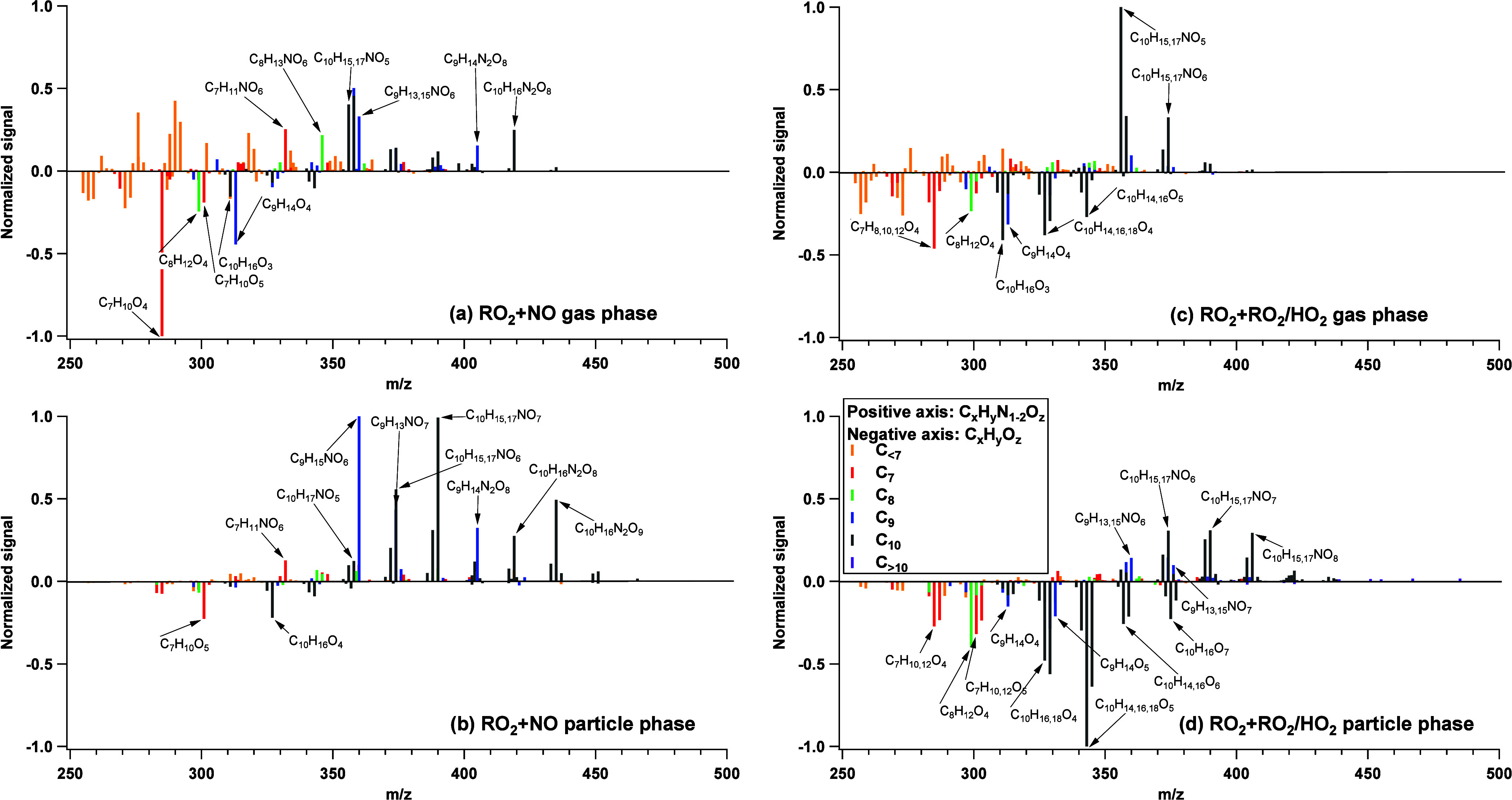
FIGAERO–CIMS mass spectra of all gas- and particle-phase products under different experimental conditions for 3°_ApHN (peak SOA mass concentration cycle at time = 140–150 min after lights on): (a) gas phase in RO_2_ + NO dominant experiment, (b) particle phase in RO_2_ + NO dominant experiment, (c) gas phase in RO_2_ + RO_2_/HO_2_ dominant experiment, and (d) particle phase in RO_2_ + RO_2_/HO_2_ dominant experiment. Bars are colored by the number of carbon atoms as noted in the legend. For each panel, the top portion represents C_*x*_H_*y*_N_1–2_O_*z*_ compounds whereas the bottom portion represents C_*x*_H_*y*_O_*z*_ compounds. It is noted that all species in each panel a–d are normalized to the species with the highest intensity. The major products (C > 7 and normalized signal > 0.1) are labeled with their corresponding molecular formulas.

**Figure 3:**
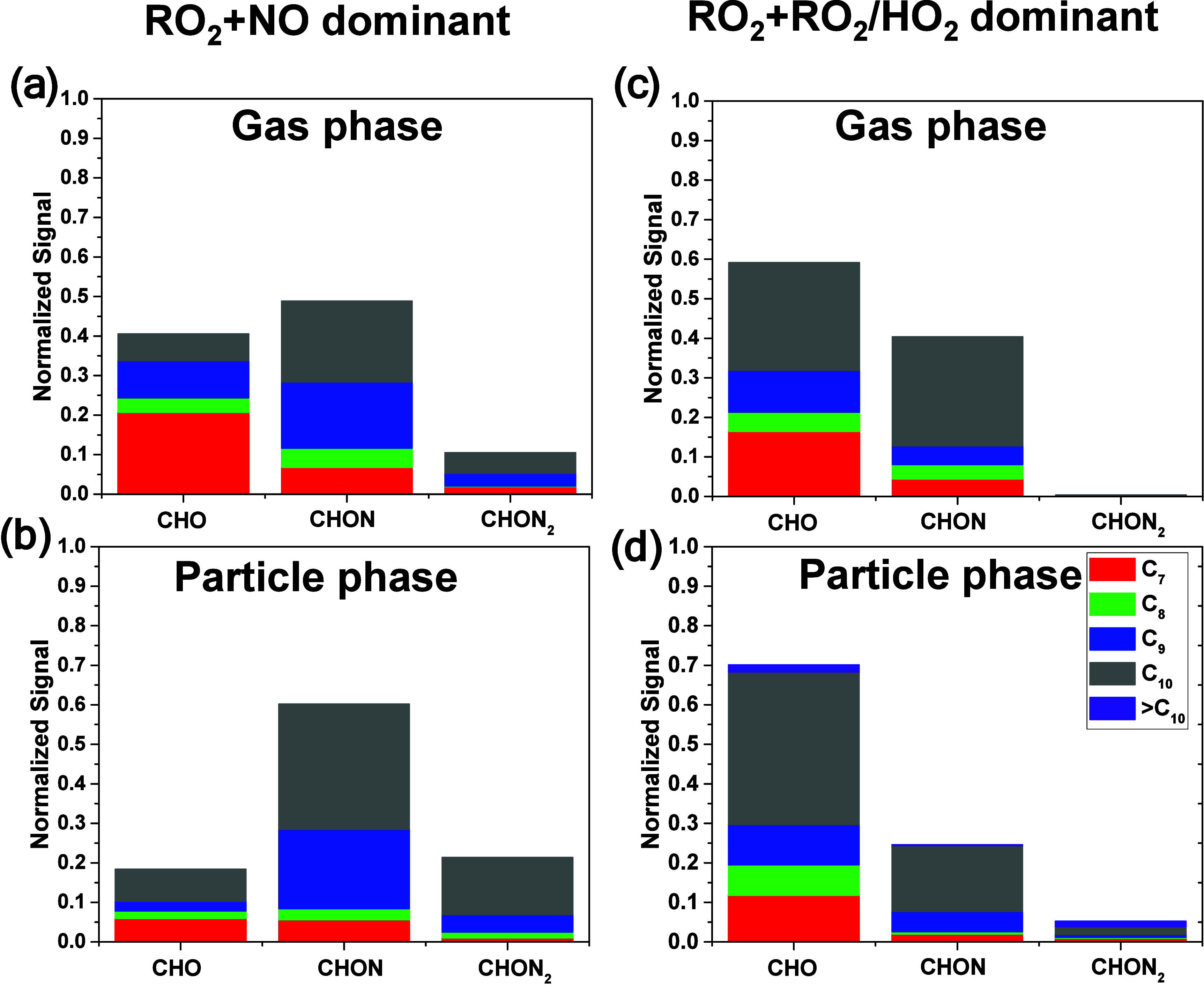
Stacked bar charts for different product families (CHO, CHON, and CHON_2_) for major products in the gas and particle phases, under different experimental conditions for 3°_ApHN (peak SOA mass concentration cycle at time = 140–150 min after lights on): (a) gas phase composition in RO_2_ + NO dominant experiment, (b) particle phase composition in RO_2_ + NO dominant experiment, (c) gas phase composition in RO_2_ + RO_2_/HO_2_ dominant experiment, and (d) particle phase composition in RO_2_ + RO_2_/HO_2_ dominant experiment. Bars are colored by the number of carbon atoms as noted in the legend.

According to previous research,^63^ OH radical can either add to a double bond or abstract a hydrogen atom, neither of which affects the nitrooxy group of MT-ONs. It is reasonable to expect that the CHON compounds would be the major products in photooxidation experiments of MT-ONs. Additionally, the subsequent reaction RONO_2_(O_2_) + NO = R­(ONO_2_)_2_ can proceed in the presence of NO to generate CHON_2_ compounds. Therefore, it is not surprising that most of the gas and particle products are CHON and CHON_2_ compounds in RO_2_ + NO dominant experiments of 3°_ApHN, 2°_LmHN, and 1°_BpHN as well as the RO_2_ + RO_2_/HO_2_ dominant experiments of 2°_LmHN and 1°_BpHN. Assuming uniform sensitivity among the detected species in FIGAERO–CIMS, 59%, 94%, and 90% of the gas-phase products, as well as 82%, 87%, and 95% of the particle-phase products, are CHON and CHON_2_ under RO_2_ + NO dominant condition for 3°_ApHN, 2°_LmHN, and 1°_BpHN, respectively. Similarly, 80% of the gas-phase and 95% of particle-phase products of 1°_BpHN, as well as 92% of the gas-phase and 89% of the particle-phase products of 2°_LmHN, still retain the nitrooxy group after photooxidation under RO_2_ + RO_2_/HO_2_ dominant condition. However, the observations are different for RO_2_ + RO_2_/HO_2_ dominant experiment of 3°_ApHN. CHON and CHON_2_ compounds contribute to only around 40% and 30% of compounds in gas and particle phases, respectively. However, it is noted that the sensitivity of FIGAERO–CIMS toward CHON and CHON_2_ species is not uniform,^65^ which can influence the observed fraction of these compounds. To address this limitation, we also employ HR-ToF-AMS measurements to provide mass-based evidence for the presence and relative abundance of ONs.

The prevalence of ON products from photooxidation of MT-ONs is also evident in the HR-ToF-AMS measurement, except for the RO_2_ + RO_2_/HO_2_ dominant experiment of 3°_ApHN, consistent with FIGARO–CIMS observation. Here, all nitrate measured by the HR-ToF-AMS are taken to be ON products. We adopt the method explained in our previous work^37,66^ and use the mass concentration ratio of particulate ONs to OA (i.e., _p_ON/OA) to calculate the fraction of ON products. It is noted that _p_ON includes both organic and nitrate components of ONs, while OA encompasses both nitrated and non-nitrated organic compounds (Table S2). The uncertainties of the _p_ON/OA ratios are calculated by propagating the statistical errors associated with the mass concentrations of _p_ON and OA measured by HR-ToF-AMS. A detailed discussion of the uncertainty analysis is provided in Section S1. In the photooxidation experiments of 1°_BpHN, the _p_ON/OA is 101.3 ± 22.6% under RO_2_ + NO dominant condition and 76.9 ± 17.1% under RO_2_ + RO_2_/HO_2_ dominant condition. Although the size distribution of SOA generated in RO_2_ + NO dominant experiment of 2°_LmHN exceeds the size range of the HR-ToF-AMS, preventing accurate calculation of _p_ON/OA, _p_ON/OA of 2°_LmHN is still fairly high at 51.0 ± 11.4% under RO_2_ + RO_2_/HO_2_ dominant condition. However, _p_ON/OA is only 15.0 ± 3.3% in RO_2_ + RO_2_/HO_2_ dominant experiment for 3°_ApHN, approximately 5 times lower than in RO_2_ + NO dominant experiment (73.4 ± 16.4%). Overall, the notably low _p_ON/OA in RO_2_ + RO_2_/HO_2_ dominant experiment of 3°_ApHN suggests a potentially different photooxidation mechanism leading to the loss of nitrooxy group. This indicates that, in contrast to typical reactions where the OH radical can add to a double bond or abstract a hydrogen atom without affecting the nitrooxy group of MT-ONs, alternative pathways may be involved (mechanisms discussed in section 3.3). It is noted that the fractions of CHON and CHON_2_ species detected by FIGAERO–CIMS and the _p_ON/OA ratios measured by HR-ToF-AMS are in reasonable agreement [differ by 6.3–38% (0.9- to 2-fold)].

Considering the formation of NO_*x*_ or nitric acid^44^ through the loss of the nitrooxy group during the photooxidation of MT-ONs, the results from both FIGAERO–CIMS and HR-ToF-AMS measurements suggest the impact on the NO_*x*_ cycle varies depending on the molecular structure of the MT-ONs and different RO_2_ chemistry. Previous work^44^ on isoprene-derived ONs under RO_2_ + RO_2_/HO_2_ dominant condition shows that the gas phase compound distribution resulting from the photooxidation of 2-methyl-1-nitrooxy-3-buten-2-ol (C5ON) consists of 57% CHON and 43% CHO. However, the compound distribution resulting from the photooxidation of another isoprene-derived ON, 3-methyl-2-nitrooxy-4-penten-3-ol (C6ON), consists of 94% CHON and 6% CHO. As CHO compounds can only be generated from the cleavage of O–N bond from the O-NO_2_ moiety,^38^ if we consider that the fraction of CHO compounds represents the fraction of ON precursor losing its nitrooxy group to form NO_*x*_ or nitric acid, these results indicate that the photooxidation of C5ON has a greater influence on NO_*x*_ recycling compared to C6ON. Similarly, 3°_ApHN under RO_2_ + RO_2_/HO_2_ dominant experiment has a greater influence on NO_*x*_ recycling compared to other MT-ONs in our work.

### 3.3 Proposed Photooxidation Mechanism of 3°_ApHN

As 3°_ApHN (C_10_H_17_NO_4_) is the first-generation product from α-pinene photooxidation in the presence of NO,^46^ and it has a potentially different photooxidation mechanism leading to the loss of nitrooxy group, we take 3°_ApHN as an example to illustrate the photooxidation mechanism. We propose photooxidation mechanisms for the formation of the major gas- and particle-phase products shown in Figure 2. As with alkenes, reaction occurs almost exclusively by addition of the OH radical to the double bond.^67,68^ Therefore, we first propose the mechanism with OH addition to 3°_ApHN (Figure 4 and Figure S6). It is expected that the OH radical will predominantly add to the less substituted carbon atom (the C7 position in 3°_ApHN, as shown in Figure 4) in the double bond of 3°_ApHN, forming the peroxy radical C7RO2 (via reaction R2). Simultaneously, the OH radical can also add to the C2 position in 3°_ApHN (Figure 4) to produce the peroxy radical C2RO2 (via reaction R1), albeit with a minor branching ratio. Although the types of products formed in subsequent reactions depend on the molecular structures of C2RO2 and C7RO2, C_7_H_12_O_4,5_, C_10_H_18_O_4,5_, C_10_H_17_NO_8_, and C_10_H_18_N_2_O_8_ are common products observed in reactions from both C2RO2 and C7RO2. On the other hand, C_9_H_15_NO_6_ and C_10_H_17_NO_7_ are observed to be produced only from C7RO2; C_10_H_17_NO_6_ and C_8_H_13_NO_6_ are unique to reactions from C2RO2. C2RO2 and C7RO2 can either react with NO to terminate the reaction by adding the nitrooxy group to produce C_10_H_18_N_2_O_8_ or react with RO_2_ and NO to generate new alkoxy radical (RO). These alkoxy radicals formed from C2RO2 and C7RO2 can undergo either decomposition of carbon chain, followed by several steps to produce compounds with fewer than 10 carbon atoms (i.e., C_9_H_15_NO_6_ and C_8_H_13_NO_6_) or decomposition of cyclohexyl carbon skeleton, followed by several steps to produce acyclic aliphatic ONs with more oxygen numbers (i.e., C_10_H_17_NO_7_ and C_10_H_17_NO_6_) (Figure S6). In addition, these alkoxy radicals can undergo H-migration to shift the radical to C5 position in 3°_ApHN. The termination of the radical reactions can occur either by generating the peroxide (i.e., C_10_H_18_O_4_) via radical substitution with the accompanying release of NO_2_ ^69^ or decomposition to produce C_7_H_12_O_4_. Similarly, C_10_H_18_O_5_ and C_7_H_12_O_5_ are generated through the same radical termination reactions following H-migration of both C2RO2 and C7RO2. H-migration of C2RO2 and C7RO2 followed by RO_2_ + RO_2_, RO_2_ + HO_2_, RO_2_ + NO reactions or O_2_ termination can also result in the formation of C_10_H_17_NO_8_.

**Figure 4:**
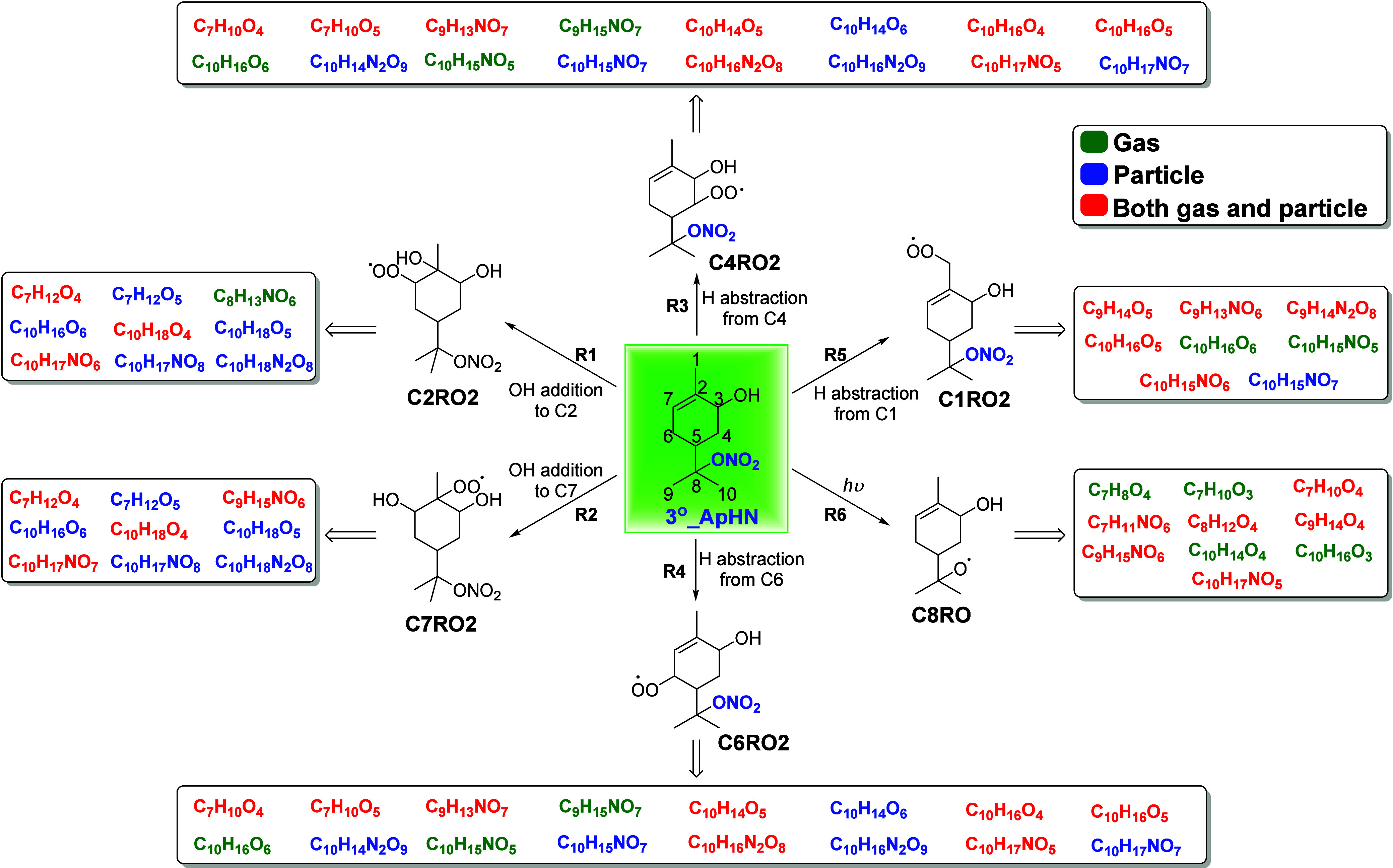
Summary of the major products formed from each pathway during the photooxidation of 3°_ApHN. Molecular formula in different colors are major products detected by FIGAERO–CIMS. The color code of products indicates different phases (i.e., whether products are detected in the gas phase only, particle phase only, or both phases), as denoted in the legend. Compounds shown in black represent the first-generation RO_2_ or alkoxyl radical from each pathway. Detailed mechanisms and the structures of each major product from each pathway are illustrated in Figures S6–S10.

As shown in Figure 2, a total of 36 compounds are detected by FIGAERO–CIMS as major products from photooxidation of 3°_ApHN in both gas and particle phases. However, OH addition to 3°_ApHN can only explain the formation of 28% of the products (i.e., 10 compounds). Recent studies^70,71^ have combined experimental approaches and theoretical calculations to demonstrate that the H abstraction pathway in monoterpene photooxidation is more important than previously thought. Specifically, it has been shown that this pathway can contribute to the formation of various products, especially highly oxygenated organic molecules (HOMs). In this work, we also consider the H abstraction pathway for 3°_ApHN photooxidation, as illustrated in Figure 4 and Figures S7–S9. In our proposed mechanisms, the OH radical can abstract the H atom from the C4, C6, and C1 positions in 3°_ApHN to produce peroxy radicals C4RO2, C6RO2, and C1RO2 via reactions R3-R5, respectively. This is because the H atom on the carbon adjacent to either a double bond or hydroxyl group becomes more reactive, promoting H abstraction. Additionally, the presence of a double bond or hydroxyl group adjacent to the C4, C6, and C1 positions in 3°_ApHN can also stabilize the resulting C4RO2, C6RO2, and C1RO2.^72^ After the formation of peroxy radical, the subsequent reactions are similar to those previously mentioned. Possible reactions include RO_2_ + RO_2_, RO_2_ + NO, RO_2_ + HO_2_, H-migration of RO_2_, decomposition of alkoxy radical, H-migration of alkoxy radical, radical substitution with the accompanying release of NO_2_, etc. Specifically, H abstraction does not affect the double bond, which can further allow the addition of radicals to that double bond. This includes processes such as RO_2_ isomerization and alkoxy radical isomerization, leading to the formation of compounds featuring cyclic structures bridged with oxygen, as well as OH addition. The products from H abstraction exhibit a higher degree of unsaturation (DBE) compared to those from OH addition, with molecular formulas like C_10_H_15_NO_*x*_, C_9_H_13_NO_*x*_, C_10_H_14/16_N_2_O_*x*_, C_10_H_14/16_O_*x*_, C_7_H_10_O_*x*_ (*x* ≥ 5), etc. Overall, the H abstraction pathway can account for 53% (i.e., 19 compounds) of the major compounds.

Figure S11 shows the time evolutions of CHO and CHON products resulting from H abstraction and OH addition, according to our proposed mechanisms (Figure 4 and Figures S6–S9). The ratio of gas-phase CHO products resulting from H abstraction to OH addition increases from 0.6 to 2.9 over time. This may suggest that CHO compounds from OH addition can further undergo H abstraction in the gas phase. For example, we find that C_10_H_16_O_6_ can be generated through H abstraction from C_10_H_18_O_5_, which is a product of OH addition (Figure S6). For gas-phase CHON compounds, the ratio of H abstraction to OH addition stabilizes at 2.1. In previous studies, the photooxidation rate constants of carbonyl ONs without a double bond (i.e., α-nitrooxyacetone, nitrooxy-butanone, nitrooxy-pentanone, and methyl-nitrooxy-butanone), which can only undergo H abstraction, are found to be much slower than those ONs with a double bond^40–44^ (Table 2). This leads to the conclusion that the double bond, considered to mainly undergo OH addition, is a major structural feature influencing the photooxidation process of ONs. However, the observation of higher levels of products from H abstraction in our study suggests that H abstraction is not a minor pathway compared to OH addition. Given that the H atom adjacent to the double bond is reactive and that the presence of a double bond can stabilize the radical to promote H abstraction,^72^ H abstraction can be considered an important photooxidation pathway only when the precursors contain at least one double bond.^70,71,73^ More theoretical calculations and experimental studies are needed to constrain the branching ratio between H abstraction and OH addition.

Additionally, C_7_H_10_O_3/4_, C_7_H_8_O_4_, C_7_H_11_NO_6_, C_8_H_12_O_4_, C_9_H_14_O_4_, C_9_H_15_NO_6_, C_10_H_16_O_3_, C_10_H_14_O_4_, and C_10_H_17_NO_5_ can be generated from photolysis of 3°_ApHN, building on findings from our prior photolysis study^34^ (Figure S10). The remaining 19% of the major compounds (i.e., 7 compounds) can be attributed to the photolysis pathway. Previous work identified C_8_H_12_O_4_, C_9_H_14_O_4_, and C_7_H_10_O_4_ are major gas and/or particle-phase products of the α-pinene photooxidation system in both RO_2_ + NO and RO_2_ + RO_2_/HO_2_ conditions. Specifically, C_8_H_12_O_4_ is identified as norpinic acid,^74^ C_9_H_14_O_4_ as pinic acid,^74^ and C_7_H_10_O_4_ as terebic acid.^75^ Our work provides additional insights into the possible formation pathways of these compounds.

## 4 Atmospheric Implications

Photooxidation initiated by the OH radical is the dominant oxidation pathway of daytime atmospheric chemistry.^45^ Previous studies have demonstrated that photooxidation is the major sink for isoprene-derived ONs and other ONs with shorter carbon chains.^40–44^ In this work, we conduct a series of chamber experiments to study photooxidation of MT-ONs (i.e., 3°_ApHN, 2°_LmHN, and 1°_BpHN), which addresses a current gap in our understanding MT-ON chemistry. The photooxidation rate constants are determined to be *k*_OH_ = (11.0 ± 1.5) × 10^–11^ cm^3^ molecule^–1^ s^–1^ for 3°_ApHN, (7.2 ± 0.4) × 10^–11^ cm^3^ molecule^–1^ s^–1^ for 2°_LmHN, and (5.7 ± 0.5) × 10^–11^ cm^3^ molecule^–1^ s^–1^ for 1°_BpHN. When considering an ambient relevant OH concentration as 1.5 × 10^6^ molecules cm^–3^, the corresponding lifetimes are 1.7, 2.6, and 3.2 h, respectively. By comparing the photooxidation rate constants of ONs from prior studies,^40–44^ SARs, and our work, we find that the presence of double bond is the key factor that controls the photooxidation rate constant. In addition, the type of functional group adjacent to the double bond also affects the rate constants. For example, a nitrooxy group adjacent to the double bond can inhibit photooxidation (e.g., 1°_BpHN), while a hydroxy group in the same position can promote photooxidation (e.g., 3°_ApHN). The measured MT-ON rate constants are similar to those estimated by SARs (within 10–30%),^63^ indicating that SARs is a useful approach in estimating photooxidation rate constants.

In addition to photooxidation, MT-ONs can undergo other loss pathways such as photolysis and ozonolysis. Through a series of systematic chamber experiments reported in this work and our previous study,^34^ we present the rate constants for photooxidation, photolysis, and ozonolysis of MT-ONs (i.e., 3°_ApHN, 2°_LmHN, and 1°_BpHN) (Figure 5 and Table S3). The lifetimes for photolysis and ozonolysis ([O_3_] = 50 ppb) of these MT-ONs range from 2 to 6.8 h and 5.3 to 20 h, respectively. The photooxidation, photolysis, and ozonolysis pathways of these MT-ONs are comparable under ambient conditions, based on experimentally determined rate constants.

**Figure 5:**
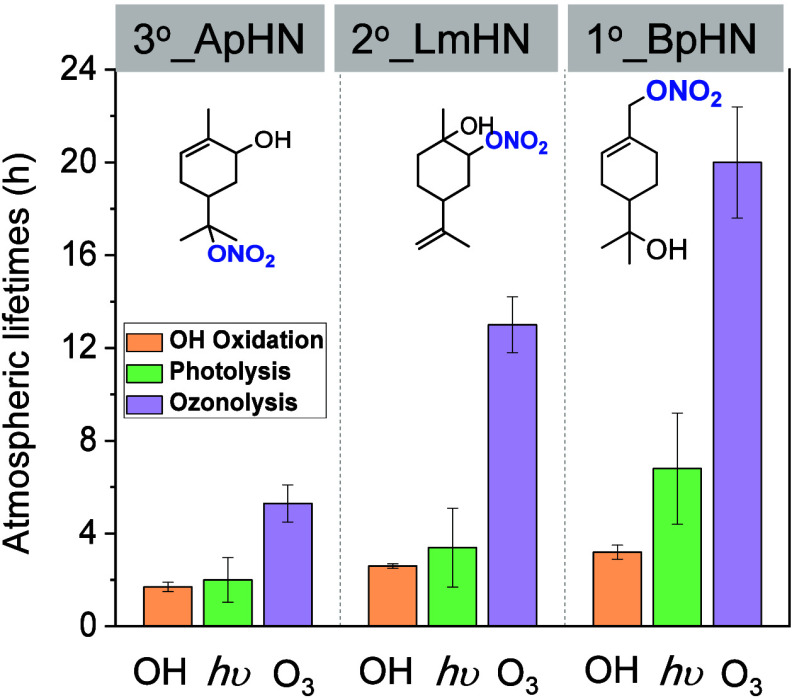
Comparison of atmospheric lifetimes of OH-initiated photooxidation, ozonolysis, and photolysis of three MT-ONs in this work and our previous work.^34^ The atmospheric lifetimes for OH oxidation and ozonolysis are estimated assuming an ambient OH concentration of 1.5 × 10^6^ molecules cm^–3^ and an ozone concentration of 50 ppb, respectively. Photolysis lifetimes are calculated using the solar spectral photon flux obtained from the TUV radiation model under the following conditions: solar zenith angle of 28.14°, solar noon on August 1 at a latitude of 33.75°N (Atlanta), an overhead ozone column of 300 Dobson units, and a surface albedo of 0.1.

The treatment of photooxidation, photolysis, and ozonolysis of MT-ONs in current chemical transport models simply relies on the estimated rate constants obtained through predictive models (e.g., Estimation Program Interface (EPI) Suite or SARs) or analogizing with structurally similar compounds. For example, although the photooxidation rate constants of MT-ONs used in Browne et al.^76^ align with the experimental values reported in this study, the photolysis rate constants for MT-ONs are estimated to be one-third of those for *tert*-butyl nitrate, which is 5–42 times smaller than our previously reported photolysis rate constants for MT-ONs under the same solar spectral photon flux.^38^ Furthermore, the ozonolysis rate constants estimated by EPI Suite are 4–15 times larger than experimental data.^38^ Overall, it is reported that photooxidation is the primary loss pathway for MT-ONs, accounting for 48% of the total losses. Ozonolysis contributes 33%, while photolysis accounts for only 3% of the MT-ONs losses.^76^ The discrepancies between modeled predictions and experimental findings for MT-ONs degradation highlight the importance of incorporating experimentally derived rate constants in future modeling frameworks to accurately simulate the atmospheric processing of MT-ONs in regional and global transport models.

Additionally, unlike the model simulations^76,77^ that regard photooxidation as a permanent sink of MT-ONs leading to their complete degradation into NO_*x*_ or nitric acid, our study offers a different perspective on this process. If we consider that the fraction of CHO compounds represents the fraction of ON precursor losing its nitrooxy group to form NO_*x*_ or nitric acid,^44^ the impact of MT-ON photooxidation on the NO_*x*_ cycle varies depending on their molecular structures and RO_2_ chemistry. Under RO_2_ + NO dominant condition, 18–41%, 6–13%, and 0–10% of gas-phase products from 3°_ApHN, 2°_LmHN, and 1°_BpHN photooxidation, respectively, do not contain nitrooxy group. Under RO_2_ + RO_2_/HO_2_ dominant condition, the fraction of CHO compounds increases, contributing 60–85%, 8–49%, and 5–23% of gas-phase products for 3°_ApHN, 2°_LmHN, and 1°_BpHN, respectively. In our chamber experiments, only a limited number of OH-initiated photooxidation steps may occur, which could limit the extent of molecular fragmentation and the potential release of NO_*x*_. However, in the ambient atmosphere, further multigenerational oxidation and the broader diversity of ONs can significantly influence NO_*x*_ recycling. Therefore, atmospheric photooxidation of ONs may play a more important role in enhancing NO_*x*_ recycling. In addition, while the RO_2_ + RO_2_ pathway may impact product distributions and the extent of NO_*x*_ recycling, explicit simulations of this chemistry remain uncertain as MT-ON oxidation is not yet included in the MCM. Therefore, incorporating more comprehensive RO_2_ chemistry in future studies is essential for better quantifying the atmospheric implications of MT-ON oxidation.

Given the limited number of MT-ONs studied, it is difficult to generalize the dominant structural features that control the role of MT-ON photooxidation in NO_*x*_ recycling. However, in our proposed mechanisms, CHO compounds can only be generated through three distinct pathways: (1) radical substitution accompanied by the release of NO_2_, directly converting the radical into peroxide (e.g., C_10_H_*y*_O_*x*_), (2) decomposition of alkoxy radicals, resulting in the loss of the carbon chain and nitrooxy group to produce compounds with fewer than 10 carbon atoms (i.e., C_<10_H_*y*_O_*x*_) and nitric acid, and (3) photolysis of MT-ONs to produce NO_2_ directly, which is challenging to distinguish from photooxidation in both chamber experiments and field observations. Further work is warranted to fully constrain the importance of each pathway and the structural features or functional groups in MT-ONs that most impact NO_*x*_ recycling during photooxidation.

## Supplementary Material



## Notes

Additional details, including calculations of MT-ON photooxidation rate constants, _p_ON/OA ratio, and experimental profiles of typical MT-ON photooxidation experiments, product distributions from photooxidation of 1°_BpHN and 2°_LmHN, proposed formation mechanisms of major products during the 3°_ApHN photooxidation experiment and ratios of products from H abstraction and OH addition, and chamber experiment data, available online at the Index of Chamber Atmospheric Research in the United States (ICARUS, https://icarus.ucdavis.edu/) (PDF)
