# Supplementary material for: OH-Initiated Photooxidation of Gas-Phase Atmospherically Relevant Monoterpene-Derived Organic Nitrates
Source: Environ Sci Technol. 2025 Dec 24;60(1):847–59. doi: 10.1021/acs.est.5c07271 (PMC12810238; doi:10.1021/acs.est.5c07271)
Supplement: Supplementary file 1 [file es5c07271_si_001.pdf]

Supplementary Information

OH-initiated Photooxidation of Gas-Phase Atmospherically Relevant Monoterpene-derived Organic Nitrates

Yuchen Wang[1,2], Yu Kang Xie[1], Masayuki Takeuchi[3], Gamze Eris[2], Nga L. Ng*[,2,3,4]

[1] College of Environmental Science and Engineering, Hunan University, Changsha, Hunan, 410082, China
[2] School of Chemical and Bimolecular Engineering, Georgia Institute of Technology, Atlanta, Georgia 30332, USA
[3] School of Civil and Environmental Engineering, Georgia Institute of Technology, Atlanta, Georgia 30332, USA
[4] School of Earth and Atmospheric Sciences, Georgia Institute of Technology, Atlanta, Georgia 30332, USA
*Corresponding Author: Nga L. Ng (ng@chbe.gatech.edu)

This supplemental information contains 3 tables and 11 figures, totaling 17 pages including the cover page.

Section S1. Uncertainty analysis of mass concentrations of species measured by the HR-ToF-AMS.

In this work, we adopt the method explained in our previous work[1,2] and use the mass concentration ratio of particulate ONs to OA (i.e., $_{p}$ON/OA) to calculate the fraction of ON products. It is noted that $_{p}$ON refers to the total mass concentration of particulate ONs, encompassing both the organic and nitrate components of the ON compounds. Similarly, OA represents the total mass concentration of organic aerosols, which includes both nitrated and non-nitrated organic compounds.

$$\frac{{}_{p}ON}{OA} = \left(\frac{NO_3}{Organic+NO_3}\right) \times \left(\frac{MW_{\ pON}}{MW_{NO_{2,ON}}}\right) \qquad \text{Eq. S1}$$

Where MW_{pON} refers to the average molecular weight of $_{p}$ON estimated from FIGAERTO-CIMS data (Table S2). $MW_{NO2,ON}$ is the molecular weight of the nitrogen-containing moiety of ONs (i.e., NO_2, 46 g mol^{-1}) measured by the HR-ToF-AMS, as discussed in detail in a recent study by Takeuchi et al. (2024).

As listed in Table S2, the uncertainty of MW_{pON} ranges from 0.37-0.82%, 0.36-0.74%, and 0.20-1.77% for 3°_ApHN, 2°_LmHN, and 1°_BpHN, respectively. The uncertainty of MW_{pON} is derived from the standard deviation of the average molecular weights obtained from 3–4 measurement cycles of particle-phase species by FIGAERO-CIMS. For measurements of NO_3 and organic in AMS, we follow the uncertainty analysis in Takeuchi et al. (2024)[1] and in Bahreini et al. (2009)[3], in which mass concentrations of species measured by the HR-ToF-AMS have the following dependencies.

$$AMS\ mass_s \propto \frac{1}{IE_{NO_3}} \frac{1}{RIE_s} \frac{1}{CE} \frac{1}{Q} \qquad \text{Eq. S2}$$

For ionization efficiency (IE), we use the average of the relative standard deviations of the slopes obtained during routine IE calibrations, which is 2%. For relative IE (RIE), we use 10% and 20% uncertainties for the nitrogen-containing moiety and organic moiety, respectively[3]. Considering that the major compounds in this study are ONs, we adopt the uncertainty related to the collection efficiency (CE) based on the average relative standard deviation of ON standard measurements reported in Takeuchi et al. (2024)[1], which is calculated to be 9%.. For sampling flow rate (Q), the uncertainty is less than 0.5%[3]. In addition to the aforementioned uncertainties presented in Bahreini et al. (2009)[3], Using standard error propagation, the overall uncertainties associated with HR-ToF-AMS derived mass concentrations for $_{p}$ON and OA are determined to be 13.6% and 17.6%, respectively. Based on these values, the uncertainty in the $_{p}$ON/OA ratio is calculated to be 22.3%.

.

Table S1. The rate constants of MT-ONs determined for each process.

Precursor	Precursor Concentration (ppb)[a]	k_{VWL} ($\times10^{-5}$ s^{-1})[b,d]	$j_{chamber}$ ($\times10^{-5}$ s^{-1})[b,d]	k_{UV} ($\times10^{-4}$ s^{-1})[c,d]	$k_{OH}/k_{cyclohexane}$[e,f]	k_{OH} ($\times10^{-11}$ $cm^3 molecule^{-1} s^{-1}$)[d,f]
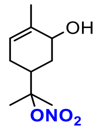 3º_ApHN	5.9 ± 0.6	1.9 ± 0.01	2.3 ± 0.8	3.7 ± 0.1	15.3 ± 2.1	11.0 ± 1.5
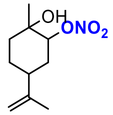 2º_LmHN	11.3 ± 0.6	0.9 ± 0.02	1.3 ± 0.5	3.0 ± 0.04	9.9 ± 0.5	7.2 ± 0.4
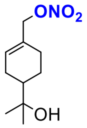 1º_BpHN	5.6 ± 0.6	1.5 ± 0.02	0.6 ± 0.1	3.1 ± 0.04	7.9 ± 0.7	5.7 ± 0.5

[a.] Measured by TD-CAPS, uncertainty = 0.6 ppb; [b.] From our previous work[4]; [c.] The uncertainties correspond to the uncertainties of linear fitting (2 standard deviations); [d.] k_{UV} = rate constant during irradiation, k_{vwl} = vapor wall loss rate constant, $j_{chamber}$ = photolysis rate constant; k_{OH}= photooxidation rate constant; [e.] Slope of concentration ln-ln plot for the photooxidation rate constant of each MT-ON relative to the rate constant of cyclohexane; [f.] The uncertainties are calculated by propagation of statistical errors associated with rate constants for the vapor wall loss, photolysis, irradiation, and slope of concentration ln-ln plot.

Table S2. The mass concentration ratio of particulate ONs ($_{p}$ON) to organic aerosol (OA) during the photooxidation of MT-ONs.

Precursor	Conditions	NO^+/NO_2^+	MW_{pON}[a]	$_{p}$ON/OA (%)[b]
3°_ApHN	RO_2+NO dominant	7.9 ± 0.1	218.7 ± 0.8	73.4 ± 16.4
	RO_2+RO_2/HO_2 dominant	7.3 ± 0.2	219.2 ± 1.8	15.0 ± 3.3
2°_LmHN	RO_2+NO dominant	4.4 ± 0.1	193.7 ± 0.7	/[c]
	RO_2+ RO_2/HO_2 dominant	5.5 ± 0.1	243.1 ± 1.8	51.0 ± 11.4
1°_BpHN	RO_2+NO dominant	5.2 ± 0.9	198.2 ± 3.5	101.3 ± 22.6
	RO_2+ RO_2/HO_2 dominant	5.3 ± 0.1	245.6 ± 0.5	76.9 ± 17.1

[a.] MW_{pON} refers to the average molecular weight of $_{p}$ON estimated from FIGAERTO-CIMS data. [b.] $_{p}$ON is the total mass concentration of particulate organic nitrate (includes the organics part and nitrate part of the ON compounds). $_{p}$ON estimated by equation: $_{p}ON = NO_3 \times \left(\frac{MW_{pON}}{MW_{NO_{2,ON}}}\right)$; $_{p}$ON/OA is calculated by the equation: $\frac{pON}{OA} = \left(\frac{NO_3}{Org+NO_3}\right) \times \left(\frac{MW_{pON}}{MW_{NO_{2,ON}}}\right) = \left(\frac{\frac{NO_3}{Org}}{1+\frac{NO_3}{Org}}\right) \times \left(\frac{MW_{pON}}{MW_{NO_{2,ON}}}\right)$. $MW_{NO_{2,ON}}$ is the molecular weight of the nitrogen-containing moiety of ONs (*i.e.*, NO_2, 46 g mol^{-1}) measured by the HR-ToF-AMS, as discussed in detail in a recent study by Takeuchi et al. (2024)[1]. [c.] The size distribution of SOA generated in RO_2+NO dominant experiment of 2°_LmHN exceeds the size range of the HR-ToF-AMS.

Table S3. Comparison of photolysis, OH-initiated photooxidation, and ozonolysis lifetimes of MT-ONs.

ONs	3°_ApHN	2°_LmHN	1°_BpHN
Structures	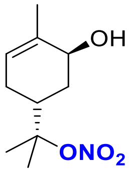	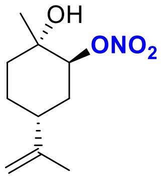	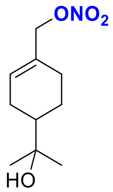
Rate constant for reaction with OH (10^{-11} cm^3 $molec^{-1}$ s^{-1})	11.0 ± 1.5[a]	7.2 ± 0.4[a]	5.7 ± 0.5[a]
OH-initiated photooxidation lifetime ([OH] = 1.5×10^6 molec cm^{-3})	1.7 ± 0.2 h	2.6 ± 0.1 h	3.2 ± 0.3 h
Rate constant for reaction with O_3 (10^{-16} cm^3 $molec^{-1}$ s^{-1})	4.2 ± 0.6[b]	1.7 ± 0.1[b]	1.1 ± 0.1[b]
Ozonolysis lifetime ([O_3] = 50 ppb)	5.3 ± 0.8 h	13 ± 1.2 h	20 ± 2.4 h
Photolysis rate constant (10^{-5} s^{-1})	14 ± 6.7[c]	8.1 ± 4.0[c]	4.1 ± 1.4[c]
Photolysis lifetime	2.0 ± 1.0 h	3.4 ± 1.7 h	6.8 ± 2.4 h

[a.] The uncertainties are propagated from the statistical errors associated with the parameters in Table S1; [b.] Data from Wang et al. (2023)[4]; [c.] Data from Wang et al. (2023)[4]. The ambient photolysis rate constants and lifetimes are calculated based on wavelength-dependent quantum yields. In addition, the solar spectral photon flux is obtained from the TUV radiation model under the following conditions: solar zenith angle of 28.14°, solar noon on August 1 at a latitude of 33.75°N (Atlanta), an overhead ozone column of 300 Dobson units, and a surface albedo of 0.1.

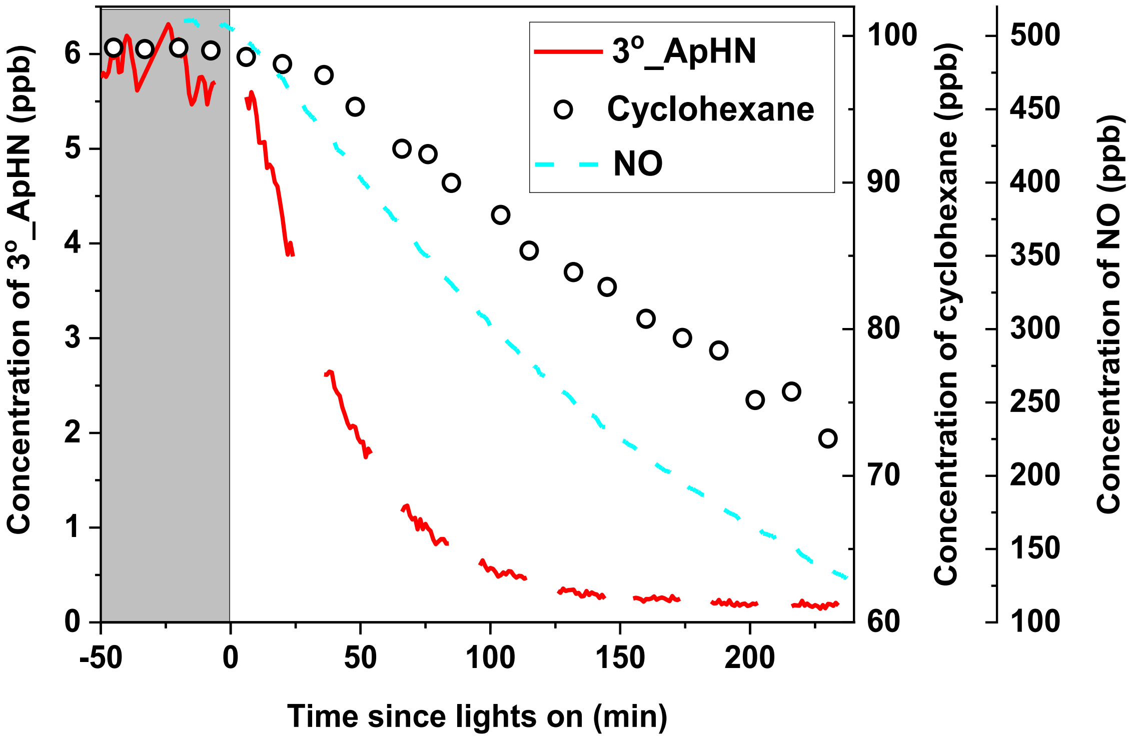

Figure S1. The time profile of a typical MT-ON photooxidation experiment using 3º_ApHN as an example. The dark shadow represents the dark period before irradiation (lights on).

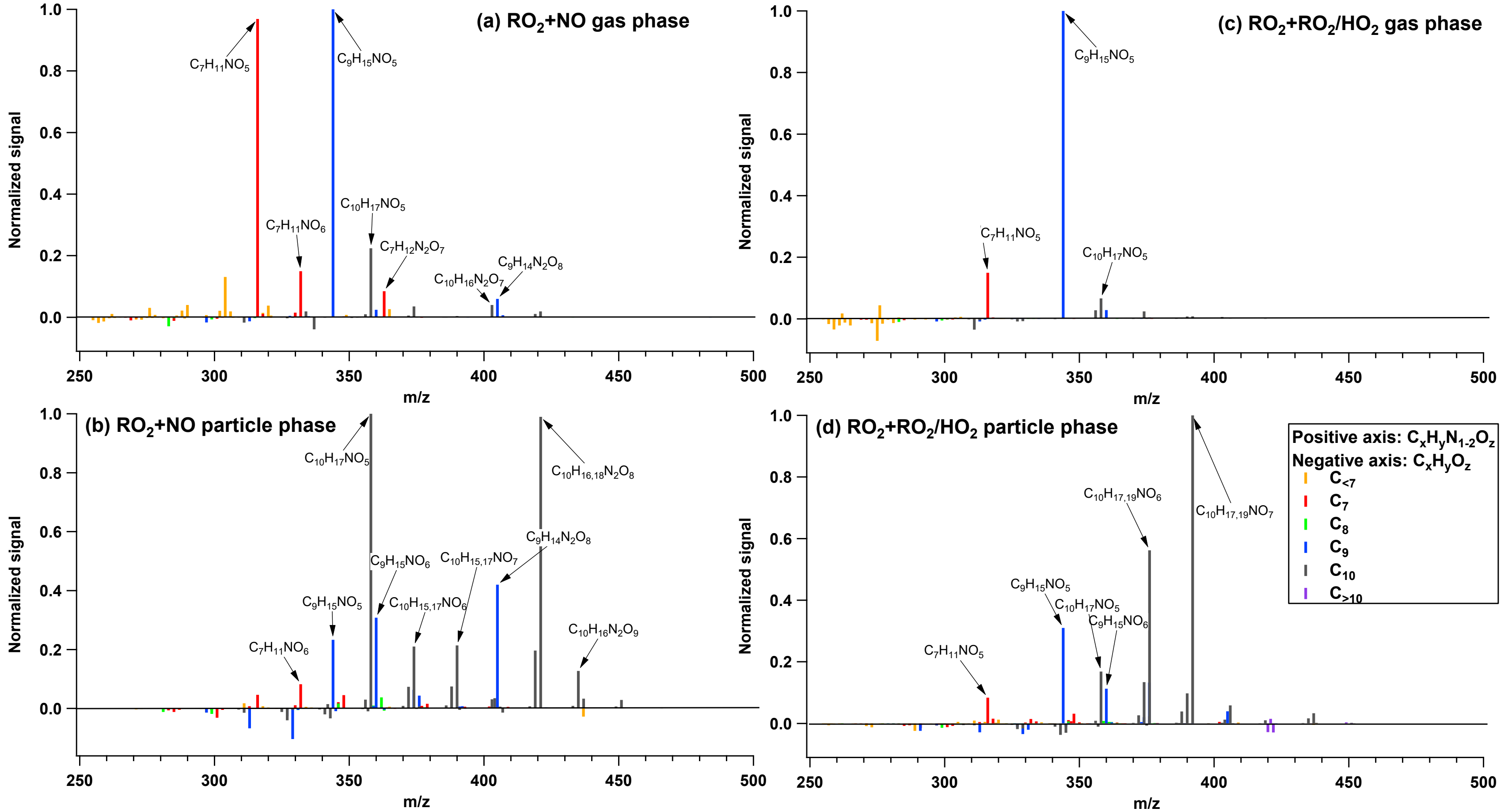

Figure S2. The FIGAERO-CIMS mass spectra of all gas- and particle-phase products under different experimental conditions for 2°_LmHN (peak SOA mass concentration cycle at time = 140–150 minute after lights on): (a). gas phase in RO_2+NO dominant experiment; (b). particle phase in RO_2+NO dominant experiment; (c). gas phase in RO_2+RO_2/HO_2 dominant experiment; (d). particle phase in RO_2+RO_2/HO_2 dominant experiment. Bars are colored by the number of carbon atoms as noted in the legend. For each panel, the top portion represents $C_xH_yN_{1-2}O_z$ compounds whereas the bottom portion represents $C_xH_yO_z$ compounds. It is noted that all species in each panel (a-d) are normalized to the species with the highest intensity. The major products (C>7 and normalized signal >0.1) are labeled with their corresponding molecular formulas.

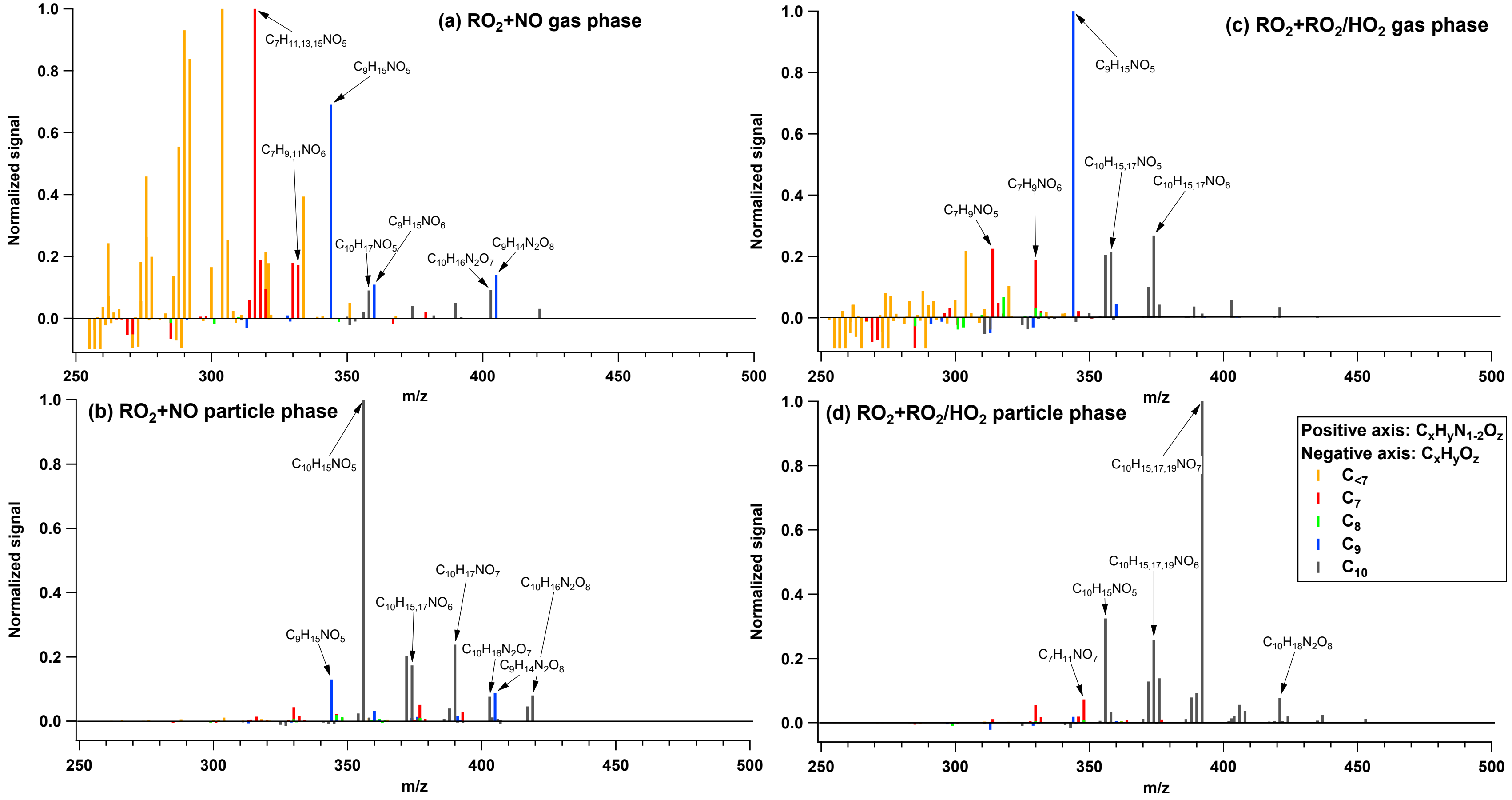

Figure S3. The FIGAERO-CIMS mass spectra of all gas- and particle-phase products under different experimental conditions for 1º_BpHN (peak SOA mass concentration cycle at time = 140–150 minute after lights on): (a). gas phase in RO_2+NO dominant experiment; (b). particle phase in RO_2+NO dominant experiment; (c). gas phase in RO_2+RO_2/HO_2 dominant experiment; (d). particle phase in RO_2+RO_2/HO_2 dominant experiment. Bars are colored by the number of carbon atoms as noted in the legend. For each panel, the top portion represents $C_xH_yN_{1-2}O_z$ compounds whereas the bottom portion represents $C_xH_yO_z$ compounds. It is noted that all species in each panel (a-d) are normalized to the species with the highest intensity. The major products (C>7 and normalized signal >0.1) are labeled with their corresponding molecular formulas.

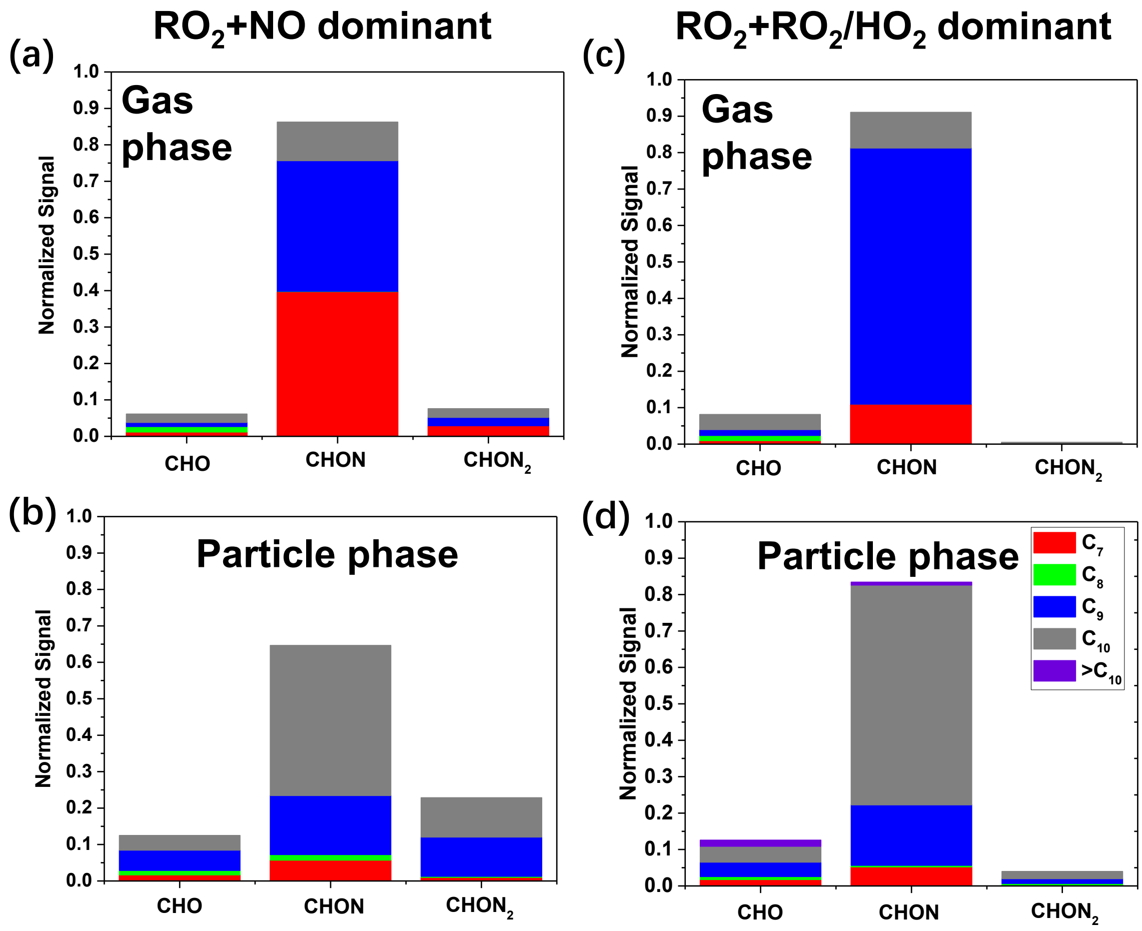

Figure S4. The stacked bar charts for different product families (CHO, CHON, and $CHON_2$) for major products in the gas and particle phases, under different experimental conditions for 2°_LmHN (peak SOA mass concentration cycle at time = 140–150 minute after lights on): (a). gas phase composition in RO_2+NO dominant experiment; (b). particle phase composition in RO_2+NO dominant experiment; (c). gas phase composition in RO_2+RO_2/HO_2 dominant experiment; (d). particle phase composition in RO_2+RO_2/HO_2 dominant experiment. Bars are colored by the number of carbon atoms as noted in the legend.

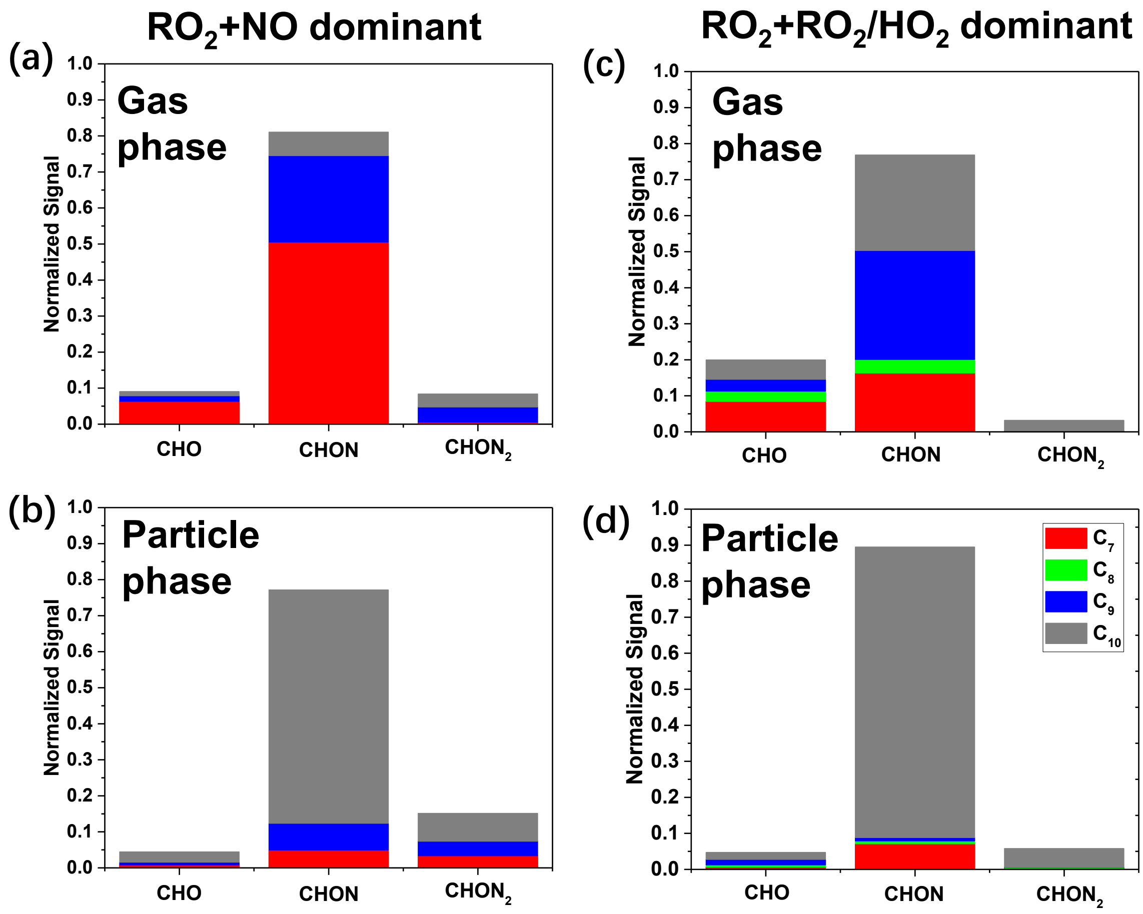

Figure S5. The stacked bar charts for different product families (CHO, CHON, and $CHON_2$) for major products in the gas and particle phases, under different experimental conditions for 1°_BpHN (peak SOA mass concentration cycle at time = 140–150 minute after lights on): (a). gas phase composition in RO_2+NO dominant experiment; (b). particle phase composition in RO_2+NO dominant experiment; (c). gas phase composition in RO_2+RO_2/HO_2 dominant experiment; (d). particle phase composition in RO_2+RO_2/HO_2 dominant experiment. Bars are colored by the number of carbon atoms as noted in the legend.

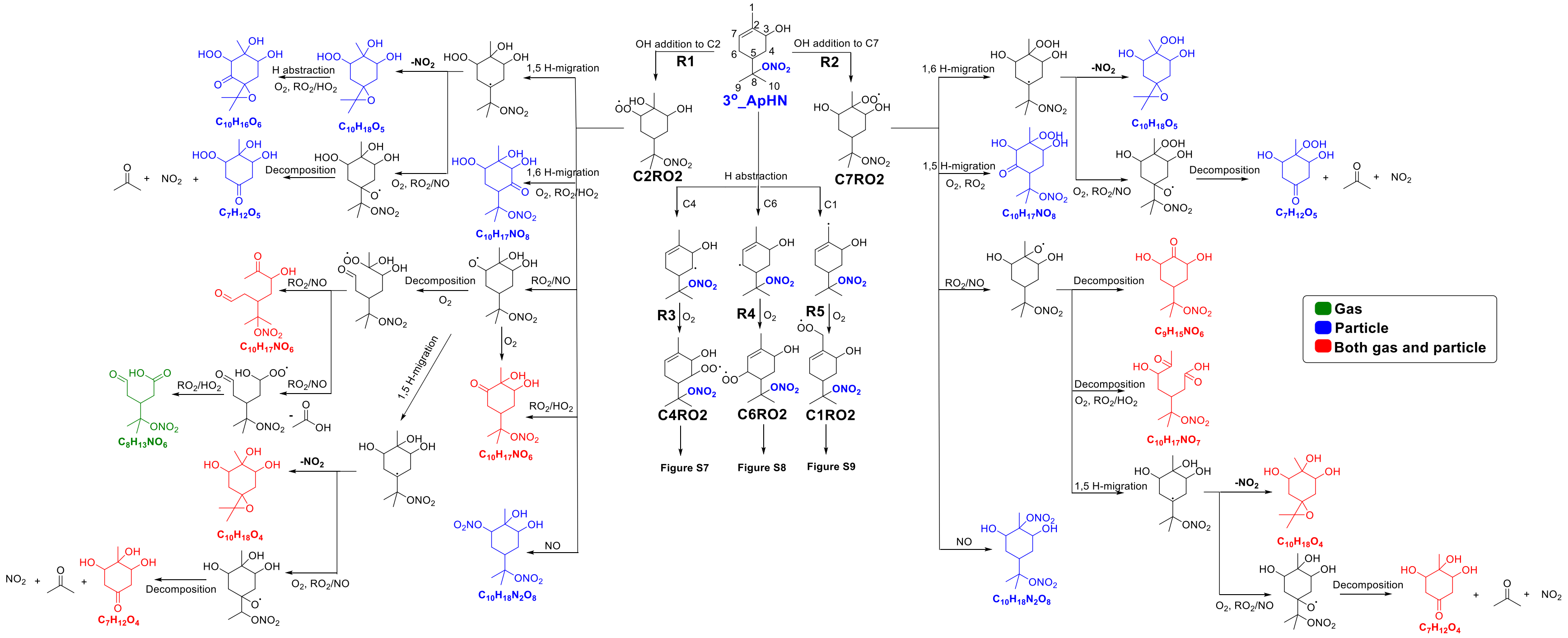

Figure S6. Proposed formation mechanism of major products during 3º_ApHN photooxidation experiment via OH addition pathway. Compounds in different colors with molecular formulas are major products detected by FIGAERO-CIMS. The color code of products indicates different phases (i.e., whether products are detected in the gas phase only, particle phase only, or both phases), as denoted in the legend. Compounds shown in black represent intermediates that are not observed.

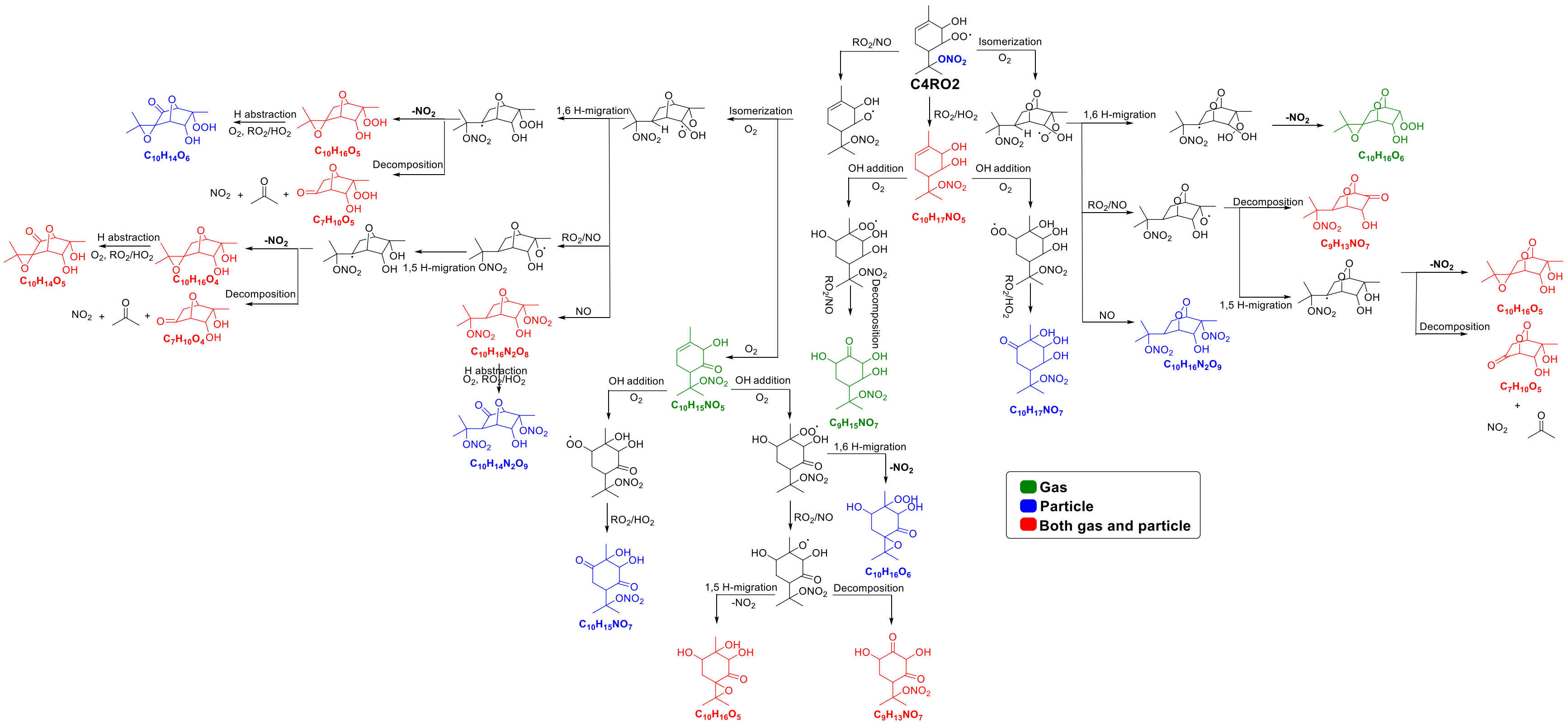

Figure S7. Proposed formation mechanism of major products during 3°_ApHN photooxidation experiment via H abstraction pathway, starting from C4RO2. Compounds in different colors with molecular formulas are major products identified by FIGAERO-CIMS. The color coding of products indicates different phases, as detailed in the legend. Compounds shown in black represent intermediates that are not observed.

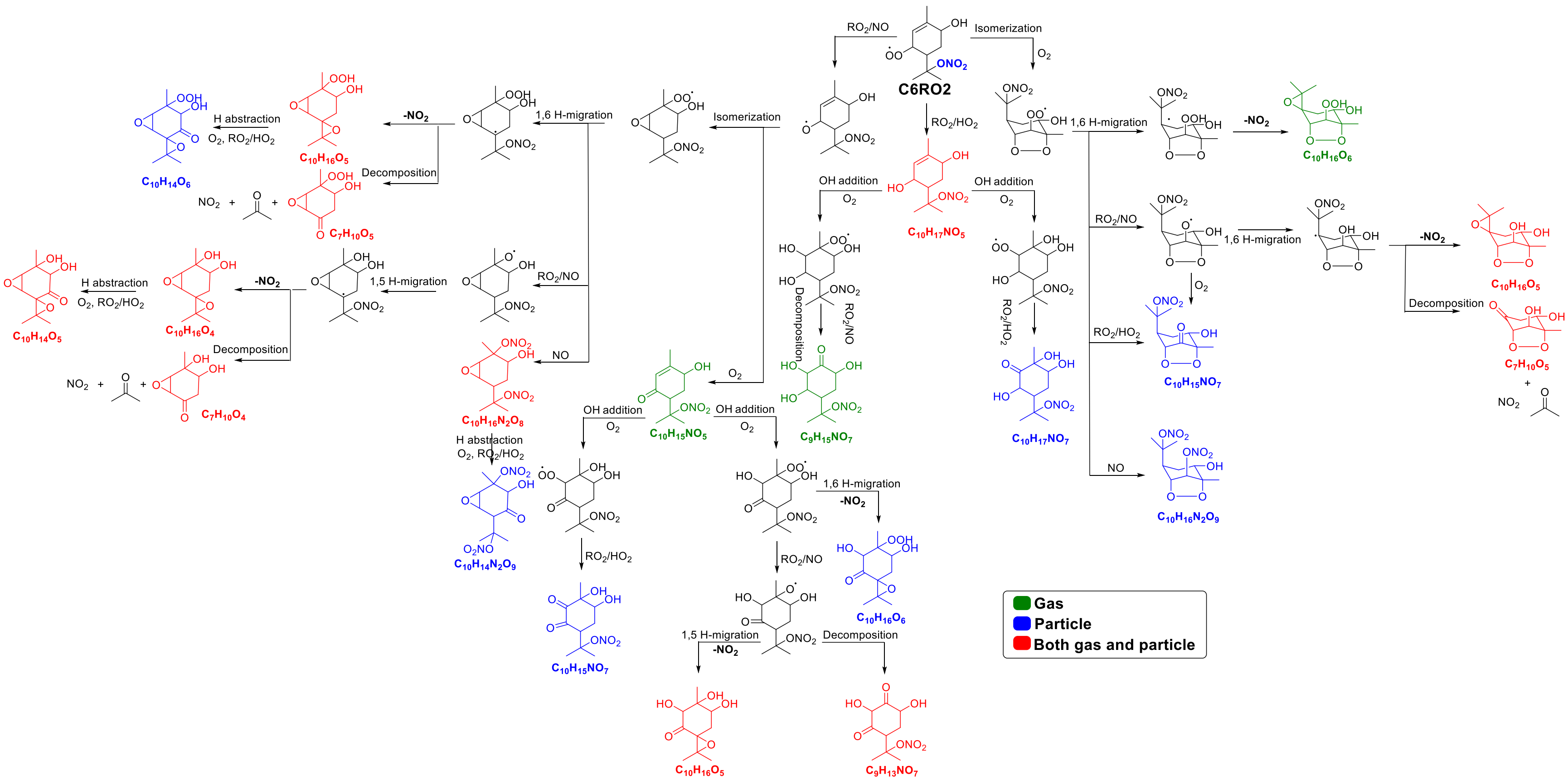

Figure S8. Proposed formation mechanism of major products during 3°_ApHN photooxidation experiment via H abstraction pathway, starting from C6RO2. Compounds in different colors with molecular formulas are major products identified by FIGAERO-CIMS. The color coding of products indicates different phases, as detailed in the legend. Compounds shown in black represent intermediates that are not observed.

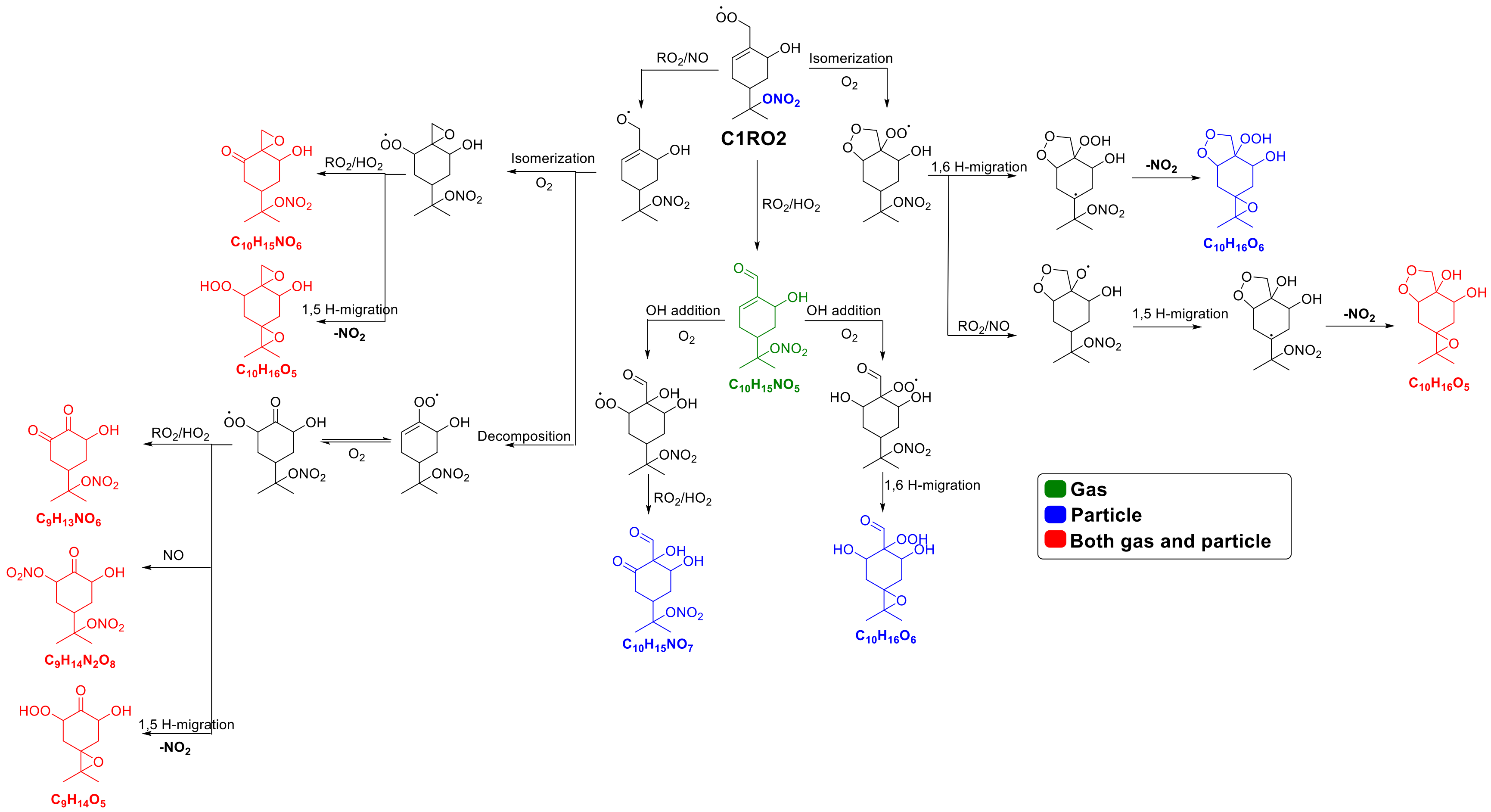

Figure S9. Proposed formation mechanism of major products during 3°_ApHN photooxidation experiment via H abstraction pathway, starting from C1RO2. Compounds in different colors with molecular formulas are major products identified by FIGAERO-CIMS. The color coding of products indicates different phases, as detailed in the legend. Compounds shown in black represent intermediates that are not observed.

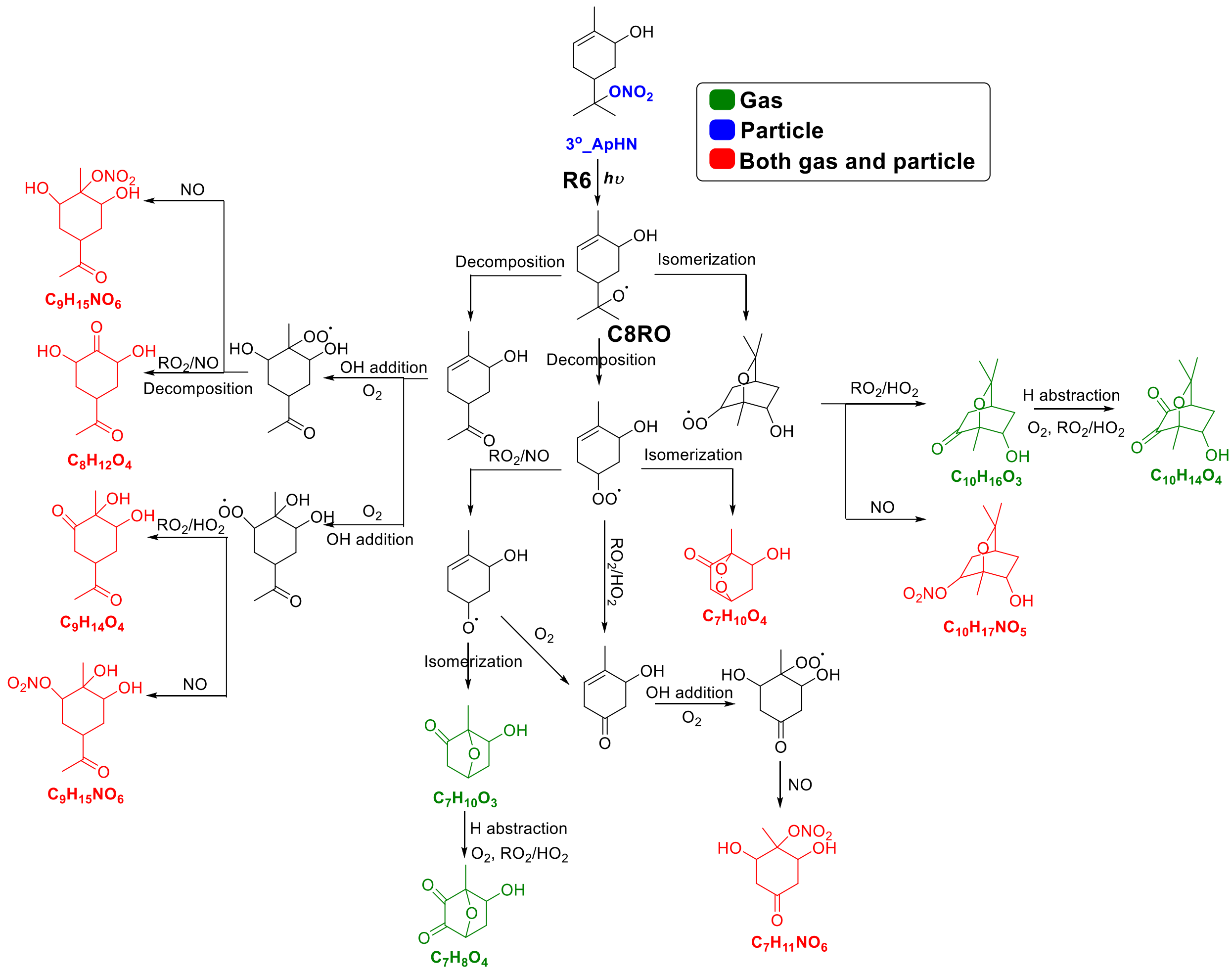

Figure S10. Proposed formation mechanism of major products from 3°_ApHN photolysis[4]. Compounds in different colors with molecular formulas are major products identified by FIGAERO-CIMS. The color coding of products indicates different phases, as detailed in the legend. Compounds shown in black represent intermediates that are not observed.

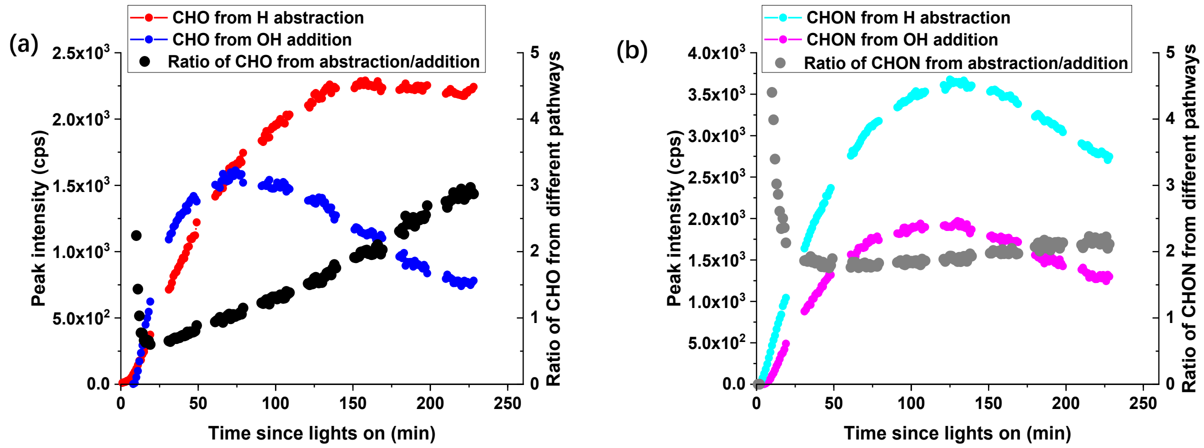

Figure S11. Time series of CHO and CHON product families resulting from OH addition and H abstraction, along with their ratios, during the photooxidation of 3°_ApHN under RO_2+RO_2/HO_2 dominant experiment: (a). gas-phase CHO products; (b). gas-phase CHON products.

References:

(1) Takeuchi, M.; Wang, Y.; Nault, B. A.; Chen, Y.; Canagaratna, M. R.; Ng, N. L. Evaluating the Response of the Aerodyne Aerosol Mass Spectrometer to Monoterpene- and Isoprene-Derived Organic Nitrate Standards. *Aerosol Sci. Technol.* **2024**, *0* (0), 1–18. https://doi.org/10.1080/02786826.2024.2389183.

(2) Takeuchi, M.; Ng, N. L. Chemical Composition and Hydrolysis of Organic Nitrate Aerosol Formed from Hydroxyl and Nitrate Rlanadical Oxidation of α-Pinene and β-Pinene. *Atmos. Chem. Phys.* **2019**, *19* (19), 12749–12766. https://doi.org/10.5194/acp-19-12749-2019.

(3) Bahreini, R.; Ervens, B.; Middlebrook, A. M.; Warneke, C.; De Gouw, J. A.; DeCarlo, P. F.; Jimenez, J. L.; Brock, C. A.; Neuman, J. A.; Ryerson, T. B.; Stark, H.; Atlas, E.; Brioude, J.; Fried, A.; Holloway, J. S.; Peischl, J.; Richter, D.; Walega, J.; Weibring, P.; Wollny, A. G.; Fehsenfeid, F. C. Organic Aerosol Formation in Urban and Industrial Plumes near Houston and Dallas, Texas. *J. Geophys. Res. Atmos.* **2009**, *114* (16), 1–17. https://doi.org/10.1029/2008JD011493.

(4) Wang, Y.; Takeuchi, M.; Wang, S.; Nizkorodov, S. A.; France, S.; Eris, G.; Ng, N. L. Photolysis of Gas-Phase Atmospherically Relevant Monoterpene-Derived Organic Nitrates. *J. Phys. Chem. A* **2023**, *127*, 987–999. https://doi.org/10.1021/acs.jpca.2c04307.
